# Imaging evolution of Cascadia slow-slip event using high-rate GPS

**DOI:** 10.1038/s41598-022-10957-8

**Published:** 2022-05-03

**Authors:** Yuji Itoh, Yosuke Aoki, Junichi Fukuda

**Affiliations:** 1 Earthquake Research Institute, The University of Tokyo, Bunkyo-ku, Tokyo, 113-0032 Japan; 2 Present Address: Institut des Sciences de la Terre, Université Grenoble Alpes, 38610 Gières, France

**Keywords:** Seismology, Tectonics

## Abstract

The slip history of short-term slow slip event (SSE) is typically inferred from daily Global Positioning System (GPS) data, which, however, cannot image the sub-daily processes, leaving the underlying mechanisms of SSEs elusive. To address the temporal resolution issue, we attempted to employ the kinematic subdaily GPS analysis, which has never been applied to SSE studies because its signal-to-noise ratio has been believed too low. By carefully post-processing sub-daily positions to remove non-tectonic position fluctuation, our 30-min kinematic data clearly exhibits the transient motion of a few mm during one Cascadia SSE. A spatiotemporal slip image by inverting the 30-min data exhibits a multi-stage evolution; it consists of an isotropic growth of SSE followed by an along-strike migration and termination within the rheologically controlled down-dip width. This transition at the slip growth mode is similar to the rupture growth of regular earthquakes, implying the presence of common mechanical factors behind the two distinct slip phenomena. The comparison with a slip inversion of the daily GPS demonstrates the current performance and limitation of the subdaily data in the SSE detection and imaging. Better understanding of the non-tectonic noise in the kinematic GPS analysis will further improve the temporal resolution of SSE.

## Introduction

Various types of geophysical observations have captured the signatures of different types of slow earthquakes, which are slow transient faulting with longer duration than regular fast earthquakes^1–3^. A slow-slip event (SSE), a class of slow earthquake, is detected by geodetic observations^4–15^. It often coincides with seismically detected slow earthquakes^5–8,16–18^, which are tremors, low-frequency earthquakes (LFEs), and very low-frequency earthquakes. In particular, the term ‘episodic tremor and slip (ETS)’ was coined to describe the coincidence of tremors and a slow slip^16^. With increasing detection capability, slow earthquakes are now considered a common fault process in many subduction zones around the world, such as Cascadia in northwestern US and western Canada^1,4–12,16,17,19,20^, Mexico^20,21^, and Nankai and Tohoku in Japan^2,13,18,22,23^, as well as continental transform boundaries such as the San Andreas Fault zone^14^. They occur in a limited depth range, typically in the deeper extension of the seismogenic zone of great regular earthquakes, due to the depth-dependent change of rheological characteristics of fault zones^1–3,24^. The intermittent occurrence of slow earthquakes adjacent to the seismogenic zone of great earthquakes has posed the question of whether they trigger great earthquakes, which is an important issue in a social context^2^. Hence, the underlying physical mechanisms of these slow earthquakes have been studied extensively with a variety of mechanisms proposed^3,25,26^.

Global Navigation Satellite System (GNSS) is one of the most powerful tools for capturing surface deformation associated with SSE^4–15,20,21,23^. Continuous GNSS position time series have been used to detect short-term SSEs lasting a few to tens of days^1,2^. Simple cumulative slip models or spatiotemporal evolution of SSE have been constructed from the extracted signature, providing observational constraints to diverse physical models of slow earthquakes^4–13,15^. However, their typical epoch-to-epoch interval is 1 day, hiding detailed slip processes of short-term SSEs with a time scale less than a day or the sub-daily processes. The paucity of subdaily resolution of data needs overcoming to understand the mechanisms of short-term SSEs and their physical relationship with the seismically detected slow earthquakes with a much shorter duration of up to hundreds of seconds^1–3,16–18^. Recent studies used tremors and LFEs as a proxy for small slips and suggested the presence of subdaily slow slip activities^15,20,21,23,27^, so robust geodetic measurement techniques to reveal the slip process in the sub-daily time scale would be most beneficial. While strainmetres and tiltmetres are sensitive to the sub-daily time scale^28^, they are not as prevalent as GNSS in many plate boundary zones, and their data suffer from local effects around the sites such as groundwater flow and precipitation^28^. These features make it difficult to extract sub-daily SSE signatures with them.

Daily static GNSS positions are deduced from a stack of raw observables of GNSS, which are the carrier phase of microwaves typically recorded at 1-, 15-, or 30-s intervals, by assuming no site motion during each day. In contrast, the kinematic GNSS analysis is a method to determine the position of sites at every raw observation epoch^29^, so it has a large potential to overcome the sampling interval issue of GNSS positions for short-term SSE studies. However, the typical noise level of the kinematic GNSS positions is in the order of centimetres^29–31^, larger than the typical short-term SSE signature^4–13^; this has prevented its application in SSE studies. With careful post-processing of kinematic positions, recent studies have successfully extracted sub-centimetre postseismic deformation^30,31^, another class of slow tectonic deformation, using kinematic GNSS positions. Motivated by these postseismic deformation studies, we attempted to apply kinematic GNSS positioning to detect and image the SSE evolution for the first time. In this study, we processed raw GPS observables recorded during a previously reported SSE in northern Cascadia from March to April 2017^9^ and inferred its spatiotemporal slip from these data. Then, we discuss tectonic implications from the inferred SSE process using the kinematic GPS. For the comparison purpose, we also derived a slip model of the same event using the daily static coordinates and discussed the performance of the kinematic GPS processing on the SSE exploration as well as its current limitations.

### Data analysis

**Figure 1:**
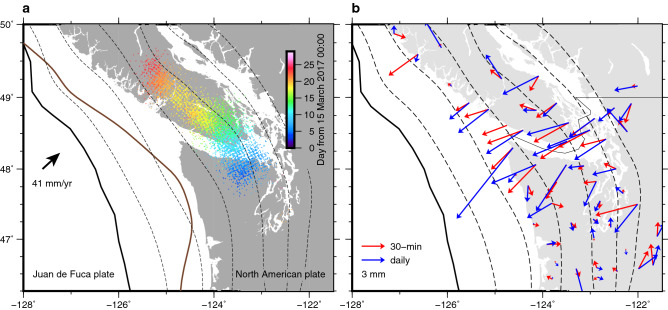
Tectonic setting of Cascadia subduction zone and comparison of cumulative GPS displacements derived from the 30-min and the daily data. (**a**) The solid bold black line indicates the trench of the two converging plates as labelled. The broken lines indicate slab surface depth contours (10 km interval from 10-km depth)^37^. The solid brown line outlines the interseismic locking depth^38^, defined as a contour of locking ratio = 0.7. The small dots color-coded with time indicate tremor epicentres detected by PNSN (https://pnsn.org/tremor; Downloaded in 25 June 2021). (**b**) Cumulative GPS displacements at selected sites using the 30-min (red) and the daily (blue) data. The cumulative displacements for the 30-min data are obtained as the difference of the initial and the final positions, which are defined as the average positions during the first (15–17 March 2017) and the last 3 days (10–12 April 2017), respectively. Outliers deviating from the average by more than 3 times of the standard deviation were removed when taking the averages. The cumulative displacements for the daily data are simply obtained by the difference of the positions at the first (15 March 2021) and the last days (12 April 2021). Prior to the displacement computation, each time series were corrected for whole network translation estimated in each slip inversion. The same figure but showing all the sites in the entire area is presented in Fig. S4.

**Figure 2:**
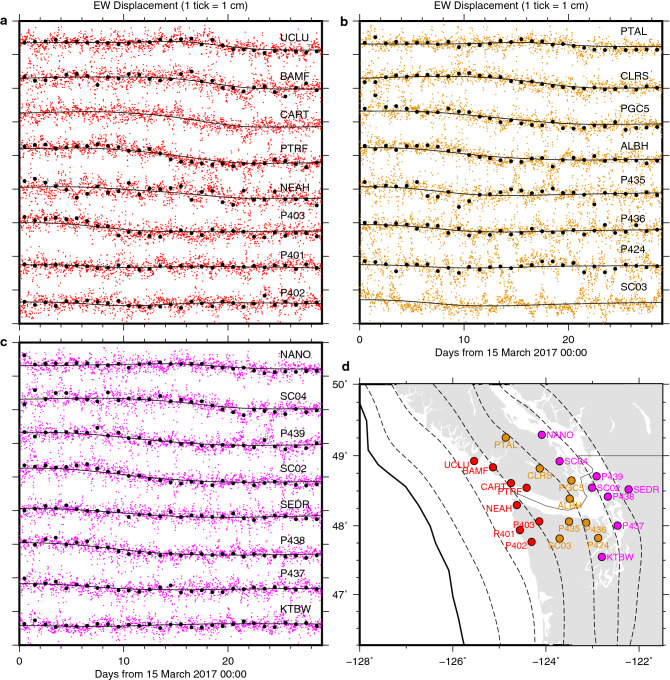
Kinematic GPS data at a 30-min interval and fitting by spatiotemporal slip model. (**a**–**c**) The coloured dots indicate the post-processed east component of kinematic 30-min GPS positions, further corrected for whole network translation estimated in 30-min slip inversion (see “Methods”). The overlying black dots indicate daily static east component which were corrected for whole network translation estimated in the daily slip inversion (see “Methods”). The daily solutions happen to be unavailable at CART and SC03. The overlying black solid lines indicate the predicted motion due to the fault slip. (**d**) Site location. The coloured circles indicate the site location of the time series with the same colour in (**a**–**c**).

**Figure 3:**
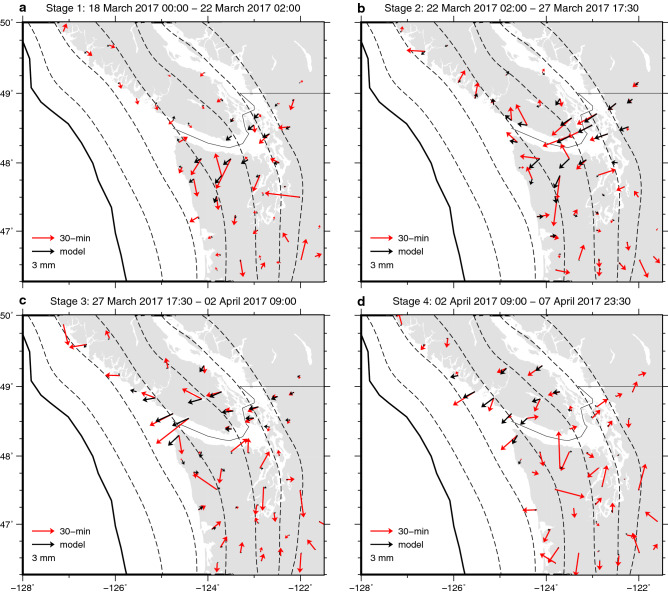
Displacements using the 30-min data (red) with the prediction of the 30-min spatiotemporal slip model (black) during four stages as labelled. The displacements in each stage for the 30-min data are obtained as the difference of the initial and the final positions, which are defined as the average positions during the 2 days centring at the first and the last epoch of each stage, respectively. Outliers deviating from the average by more than 3 times of the standard deviation were removed when taking the averages. Prior to the displacement computation, the 30-min time series were corrected for whole network translation estimated in the slip inversion. The model displacements are also obtained by the averaging using the same time window but without the outlier removal.

The ETS activity in northern Cascadia is well known to occur beneath the onshore area (Fig. 1a) where the GNSS network has been deployed (Figs. 2 and S1–S3)^4–12^. An inversion of the daily GPS data has revealed that the March to April 2017 Cascadia SSE migrated from ~ 48.0 N towards the northwest along the fault-strike with roughly concordant migration of tremors^9^. We obtained kinematic subdaily positions at sites in northern Cascadia by processing the raw GPS observables at an interval of 30 s and subsequently performed post-processing to retrieve the transient deformation signature likely due to the SSE. By correcting the 30-s kinematic coordinates for temporally^32–35^ and spatially^36^ correlated errors as well as some outliers, we finally obtained 30-min interval coordinate series at 64 sites (see “Methods”) (Fig. S3). The nominal error of these coordinates at each epoch is typically 1–2 cm for the horizontal components. Yet, at most sites, our cleaned 30-min coordinates reproduced sufficiently the trajectory of the daily static coordinates (Figs. 2 and S1, S2). They exhibit coherent transient motion to the west (Fig. 2), consistent with SSE-induced deformation in this region^4–12^. The onset of the transient signature appears to be delayed to the northwest, consistent with the along-strike SSE propagation imaged by inverting daily static GPS data during the same period^9^ as well as the propagation of tremors to the northwest (Fig. 1a), although it is not easy to visually identify a precise timing of the onset at each site. A similar transient signature is not as clearly discernible in the north component of the position time series as in the eastern component (Fig. S1). However, guided by the eastern component as well as the daily coordinates, we can discern the SSE-induced subtle transient deformation. A similar coherent transient motion or permanent offset is not discernible at sites distant from the ETS area we focus on (Fig. S2), although they exhibit fluctuations with an amplitude of ~ 5 mm. The spatial pattern of displacements supports the above-mentioned discussion (Figs. 1b and 3). The cumulative displacement patterns derived from the 30-min and the daily coordinates are well in agreement in the main ETS area, exhibiting the trenchward motion of these sites (Fig. 1b). The temporal change in the area of the main trenchward motion suggests the migration of SSE (Fig. 3). The motions of the distant sites do not exhibit such coherent pattern (Fig. S4). Hence, although the nominal error of each position is larger than the expected SSE-induced deformation amount (~ a few to several mm^4,6–12^), we concluded that our sub-daily GPS positions successfully captured previously reported transient motion associated with the March to April 2017 SSE with a finer sampling interval.

### Spatiotemporal slip inversion using subdaily data

**Figure 4:**
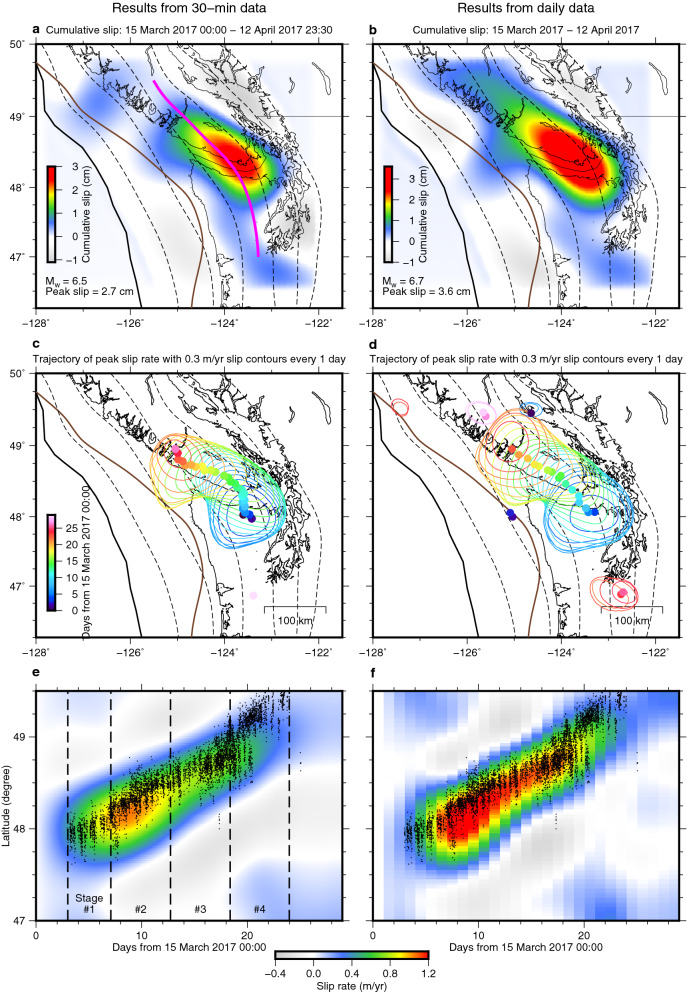
Comparison of inversion results using 30-min and daily data. Left (**a**,**c**,**e**) and right (**b**,**d**,**f**) columns indicate results from inversions of 30-min kinematic and daily static GPS positions, respectively. (**a**,**b**) Cumulative slip of the March to April 2017 SSE. The thick purple curve in panel (**a**) indicates the location of the profile for (**e**,**f**). (**c**,**d**) Collection of slip rate contours of 0.3 m/yr every day is color-coded with time. Colour-coded dots indicate the trajectory of peak slip rate location. The colour of trajectory and contours indicates the time. (**e**,**f**) Temporal evolution of slip rate along a profile along 35-km slab interface depth [the purple curve in (**a**)] with tremors (dots), epicentres of which are located above the 30- to 40-km depths slab interface.

**Figure 5:**
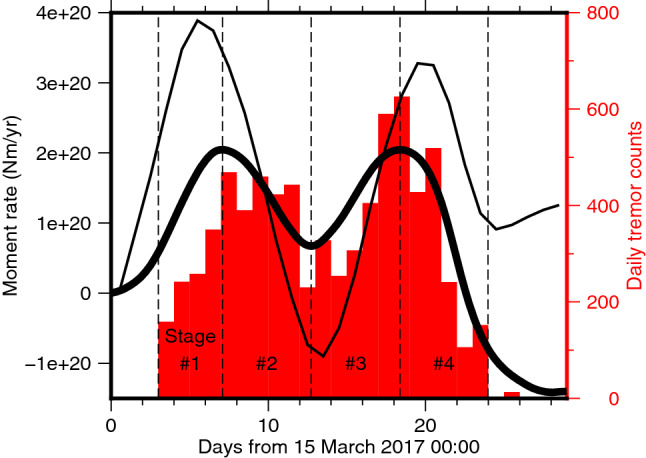
Moment rate history (solid curves) and daily tremor counts (red histogram). The thick and thin solid curves indicate moment rate history of 30-min and daily data inversions, respectively. The vertical broken lines indicate the boundaries of the four stages (Figs. 3, 4e and 6).

**Figure 6:**
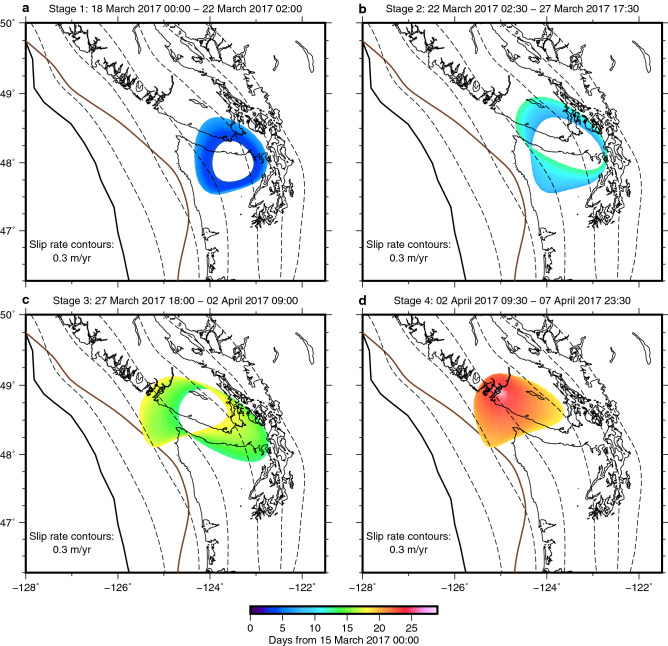
Spatiotemporal slip propagation drawn as collection of slip rate contours of the same slip rate (0.3 m/yr) during four stages as labelled. Collection of slip rate contours of 0.3 m/yr at a 30-min interval is colour-coded with time.

We performed a spatiotemporal slip inversion of the SSE using our cleaned 30-min kinematic GPS and a Kalman filter-based inversion method with a time-invariant temporal smoothness parameter^39,40^ (see “Methods”). At first, we attempted to determine the spatial and temporal smoothness parameters by maximising the log-likelihood^40^ (Fig. S5), but the obtained slip evolution was extremely oscillatory for reasons we discuss later. Hence, after testing several models with different spatial and/or temporal smoothness (Fig. S6), we present a preferred model of spatiotemporal slip propagation as a possible scenario (Figs. 4a,c,e, 5 and 6 and Videos S1 and S2) (see “Methods”) which provides the reasonable fit to the data (Figs. 2 and 3). The final moment magnitude (M_w_) for the entire period is 6.5 (Fig. 4a). The moment rate history of the SSE has two peaks in the 30-min data inversion, consistent with two peaks of daily tremor counts (Fig. 5).

We divided the inferred spatiotemporal slip evolution using the 30-min data into four stages based on the moment rate history (Fig. 5). We determined the initiation and termination of Stage 1 and Stage 4, respectively, based on daily tremor counts (Fig. 5), because our data post-processing required to assume that no SSEs occur when no tremors are detected (see “Methods”). In the first stage, our model shows slow crack-like isotropic growth of the SSE at 30–50-km depth with little migration (Stage 1; Figs. 4c and 6a and Video S2). This depth range is typical of short-term SSE occurrence in this region^1,4–6^. The 30-min data does not exhibit the clear trenchward motion pattern during this stage, which is due to the poor signal-to-noise ratio of the trench-normal component. Yet, guided by the model prediction, we can notice the moderately consistent trenchward component in the data at some sites in and around the slip area (Figs. 3a and 6a). Next, the isotropic growth is followed by an along-strike pulse-like migration towards the northwest within the depth range (Stage 2; Figs. 4c and 6b and Video S2). The along-strike migration continues and then the slip area expands to shallower depths at ~ 48.5 N, where the down-dip limit of the locked zone is also shallower. Eventually, the slip area reaches the down-dip limit of the locked zone^38^ (Stage 3; Figs. 4c and 6c and Video S2). This shallowing of the main slip area is clearly supported by the westward (i.e., up-dip) migration of the main trenchward motion area in Stages 2 and 3 (Fig. 3b,c). Finally, the slip migrates again in the along-strike direction and terminates with a blurred slip rate pattern (Stage 4; Figs. 4c, 6d and Video S2). This slip migration is also clearly supported by the 30-min data (Fig. 3c,d). Our SSE model exhibits the onset of isotropic growth slightly earlier than the onset of the tremor sequence (Figs. 4e, 5 and 6a). One possible cause for them is the time-invariant smoothness parameter for temporal slip rate (see “Methods”), as has been reported^39^. Indeed, our kinematic GPS time series exhibits a more abrupt onset of the transient surface motion than the predicted SSE-induced motion at some sites (e.g. ALBH; Fig. 2b), suggesting that the actual slip onset is more abrupt than that inferred in this study but is not well constrained.

The transition of unbounded-bounded source growth was conceptualised from observations of regular earthquakes^41^. Gomberg et al.^42^ has proposed that this growth mode transition is also the case for slow earthquakes and reconciles controversial mechanical scaling of regular and slow earthquakes^19,21,42,43^. The key idea is that slip growth is bounded (one-dimensional) or unbounded (two-dimensional) depending on whether its slip area (hence moment) is large enough to fill the limited depth range^41,24^, but direct observational evidence of the slip clearly exhibiting the unbounded-bounded growth transition is limited^6^. The inferred SSE in this study shows the multi-stage evolution, comprising the SSE growth within the limited depth range with little migration (i.e., unbounded growth in Stage 1; Figs. 4c,e and 6a and Video S2) and the subsequent unilateral migration after fully filling the depth range (bounded growth in Stages 2–4; Figs. 4c,e, and 6b–d and Video S2), which results in an elongated cumulative slip pattern in the along-strike direction (Fig. 4a and Video S1). Bletery and Nocquet^6^ already reported the similar unbounded-bounded growth transition of a different SSE from the daily GPS data in Cascadia. This study adds supportive observational evidence of the unbounded-bounded transition of the SSE growth mode with the clearer unbounded growth process. Moreover, the crack-like up-dip growth at Stage 3 coincides with the shallowing of the locking depth. The shallower locking depth is likely equivalent to the shallower up-dip boundary of the rheologically permitted SSE zone, so the up-dip growth of the slip area highlights the SSE growth mechanics controlled by fault rheology^24^. Our slip model implies the presence of common mechanical factors behind the rupture kinematics of regular and slow earthquakes, which needs testing through future investigation using the subdaily GPS data with more events.

### Relationship of the inferred SSE process and tremors

The inferred spatiotemporal slip rate evolution is also roughly concordant with the tremor migration history (Fig. 4e and Video S2), but their spatial relationship is different among the four stages. The tremors occur near the centre of growth during Stage 1, but their epicentres are in the periphery of the high slip rate area at Stage 2. The tremors and the high slip rate area overlap again during Stage 3, but the tremors cluster away from the main slip area in Stage 4. This feature implies multiple tremor excitation mechanisms during one short-term SSE, such as direct loading by slow-slip and stress increase ahead of the slip front^8,12^.

We compared the moment rate history with the daily tremor counts; both of them have consistent time evolution at this resolution (Fig. 5). Based on the sub-daily variation of tremor activity (Figs. 4e), the presence of the sub-daily variation of slow slip rates has been proposed^27^. However, our preferred slip rate model and the associated moment rate does not have such features (Figs. 4e, 5 and 6). The test model in which more vigorous slip rate change is allowed demonstrates the extremely oscillating moment rate history (Test 4 in Fig. S6e), which compelled us to impose a stronger temporal smoothing of slip rate during the entire period. However, as we further discuss in the next section, this result does not exclude the previously proposed possibility of subdaily variation of slip rates^15,20,21,23,27,28^.

### Comparison with daily data inversion and technical limitations of subdaily data

For a comparison purpose, we performed the inversion using the daily coordinates and the same method (Figs. 4b,d,f and Videos S3 and S4) (see “Methods”). Maximising the log-likelihood provided the same spatial smoothness and the slightly larger temporal smoothness parameters (i.e., rougher time evolution of slip) than the subdaily analysis, which yield a physically plausible spatiotemporal slip distribution in the daily data inversion. The cumulative slip patterns of the 30-min inversions (Fig. 4a) are qualitatively consistent with models for the same event derived from the daily static coordinate data in this study (Fig. 4b) and Michel et al.^9^, demonstrating that the noisy subdaily coordinates have the ability to reproduce realistic slip patterns. The final moment magnitude for the entire period from the 30-min data inversion (M_w_ 6.5, Fig. 4a) is slightly smaller than the estimate from the daily coordinates (M_w_ 6.7; Fig. 4b). We do not pursue the significance of the quantitative difference between the two slip models because there exist some uncertainties from the inversion parameter tuning and our insufficient understanding of the noise characteristics of the kinematic GPS, as we discuss later.

Although a moment rate history with one peak was inferred in a daily inversion model in the previous study^9^, our daily static and 30-min data inversions produced the moment rate history with two peaks (Fig. 5), so the increase of the number of the moment rate peaks originates mainly from the inversion strategy used in this study; the 30-min kinematic coordinates themselves do not have significant contribution to this improvement in temporal resolution. Nevertheless, in other words, our 30-min and daily data inversions demonstrate that the temporal resolution of the slip and moment rates derived from the 30-min coordinates can reach at least the comparable level to those derived from the daily coordinates, which is astonishing given the generally-believed higher noise level of kinematic GPS coordinates than that of daily coordinates.

We have pointed out the issue in the temporal smoothing scheme of the subdaily inversion so far, which is, to a certain extent, responsible for the comparable time resolution of subdaily and daily slip and moment rates. Yet, apart from this inversion technique issue, there exists a more important and difficult issue; it is comprehensive understanding of non-tectonic signatures contaminating the kinematic GPS positions such as tropospheric and ionospheric effects and multipath as well as other enigmatic site-specific effects^29^. Although we largely mitigated these effects during the positioning and its post-processing (Fig. S7), the failure to determine some hyperparameters in the slip inversion strongly suggests the presence of significant unmodelled non-tectonic signatures left in the kinematic data; such non-tectonic noises were formally described by the spatiotemporally rough slip distribution by exploiting the non-parametric formulation of slip and slip rate^39,40^. From the daily data, the plausible slip evolution was inferred with the hyperparameters determined by maximising the log-likelihood (See Methods), which also highlights the presence of unmodelled noise in the “cleaned” 30-min kinematic coordinates. This study has not fully addressed this issue and hence the temporal resolution of SSE inferred from the 30-min data still stays at the comparable level to the daily data. Further improvements in modelling and/or removing such noise are crucial to conclude whether we can resolve subdaily variations of slip rate and to detect isolated events lasting shorter than 1 day. At this point, this study provides the minimum recipe of the non-tectonic noise mitigation as a milestone for such future investigation.

## Methods

### GPS kinematic analysis

We determined the relative position of the GPS sites with respect to the reference site DRAO (Fig. S3) every 30 s between 11 March (day of the year (DOY) 070) and 17 April (DOY 107) 2017 using the TRACK program of GAMIT/GLOBK^44–46^ and the IGS final satellite orbit. We only employed GPS observables as those from other satellite systems (e.g., GLONASS) are not available at some sites. We used observables from GPS satellites with an elevation angle of 15° or greater. We used the Vienna Mapping Function^47^, the IGS ionosphere products (i.e. IONEX) and FES2004 tidal model^48,49^ in the centre of mass of the solid Earth to account for tropospheric, ionospheric, and tidal effects, respectively. We took a priori positions of each site on each day from the static daily solution of the Nevada Geodetic Laboratory (NGL)^50^ or, if not available, the header of each RINEX file with its uncertainty assumed to be 10 cm or 50 cm, respectively. The random-walk parameter of the position determination was set to $1 \;{\text{cm}}/\sqrt {30 \;{\text{s}}}$. First, we divided the 64 kinematic GPS sites into eight groups consisting of nearby sites and performed kinematic analysis for each group separately relative to the reference site in each group (Fig. S3). Then, we performed another kinematic analysis for the eight reference sites used in the first analysis, with respect to DRAO. Finally, we took the summation of the solutions of the two steps in the geocentric coordinate system to obtain the kinematic position relative to DRAO. We take this two-step approach to exploit the advantage of the relative positioning method, which mitigates the effects of the atmosphere on the microwave pathway more efficiently for a shorter baseline. The obtained relative position (black time series in Fig. S7a) is mostly free from the rigid plate motion of the North American plate.

### Data post-processing

We performed several steps of post-processing of the kinematic GPS coordinates to mitigate non-tectonic noise due to recognised sources in previous studies and some random outliers (Figs. S7–S15). First, we applied a sidereal filtering technique to mitigate coordinate fluctuations caused by multipath^32–35^. We assumed the repeat period of the sidereal filter to be 86,154 s^34,35^ and generated a sidereal filter by stacking six 86,154-s length time series made of those during DOY 070–072 and 104–107. The sidereal filter must be generated from the data during which no significant crustal deformation occurs^32–35^. Because no tremors were detected during these two periods by the PNSN network, we assumed that no SSE, accordingly no SSE-induced site motion, occurred during these periods and used the data during these periods to construct the filters. This assumption might be incorrect because an SSE without tremors has been reported before^7^. However, as presented in Fig. S7a, the fluctuation of our sub-daily coordinates was greatly reduced by sidereal filtering, so we accepted this assumption in this study. To obtain the 86,154-s length series and achieve a shift of time series by multiples of this length using the 30-s coordinates, we interpolated the 30-s interval series linearly. After stacking the six series, we concatenated the stacked series until it became longer than the period of interest (i.e. DOY 073–103). Then, we demeaned, detrended and decimated it to a 30-s interval. Finally, we subtracted the constructed sidereal filter from the position time series during the period of interest (red time series in Fig. S7a).

Then, we removed network-related fluctuation of coordinates, called the common-mode error (CME), mainly due to instability of the reference site and the coordinate system^36^. As its effect appears commonly at all the sites, we employed an independent component analysis (ICA)^51^, which decomposes time series into multiple spatiotemporally coherent modes and noise. Most of similar studies used ICA to pick-up some of the extracted modes representing the SSE signature for their subsequent analysis^9^. However, unlike such a standard approach, we applied ICA only to extract modes corresponding to CME from the data. In other words, we did not remove any modes other than the CME or the noise term from the input to ICA. We have two reasons for taking this strategy; (1) ICA could force to decompose the temporally variable deformation pattern due to the spatially migrating SSE of interest into multiple time-invariant spatial patterns and (2) such temporally changing signature, which is incompatible with the mode decomposition, might be left in the noise term. In this study, we carried out the CME removal twice, for which the data sets used for ICA are different; we used all the sites at the first step whereas, at the second step, we applied ICA separately to the eight subsets of the kinematic sites used in the kinematic analysis. This is because we noticed independent CMEs for each subset, which might represent the atmospheric disturbance effects in the second step of the kinematic analysis. The total number of modes to be extracted needs prescribing before applying ICA to the data. At the first step with all the sites, we prescribed 25 for the total number of extracted modes because we could not extract some modes representing CME with too small total number of modes prescribed, while some modes for CME were decomposed into spatially non-uniform patterns by gradually increasing the prescribed total number of modes. This competition provided us with a plausible prescription of the total number of modes. As a result, out of the extracted 25 modes, we identified three modes corresponding to CME (Fig. S8) which were subsequently removed from the sidereal-filtered data (blue time series in Fig. S7a). The permanent offsets are not discernible in their temporal function (Fig. S8b), so this CME removal approach does not remove the significant tectonic signature. As the second step, we applied the same ICA-based CME removal for each of the eight subsets of the kinematic sites. At this step, the total number of modes were set to the number of data components (i.e., 3 × number of sites) for simplicity. By inspecting the spatial pattern of all the decomposed modes, we identified one or zero modes (Figs. S9–S15), depending on the groups, corresponding to CME, and removed them (light blue time series in Fig. S6a). This group-specific CME removal could remove the local tectonic signature, which is the current limitation of our data analysis strategy. Nevertheless, in this study, we assumed that such a possibility was minimal because permanent offsets are not discernible in the temporal function of those modes (Figs. S9b–S15b).

We applied the post-processing explained so far to the relative position in the geocentric coordinate system to avoid undesirable effects caused by applying the CME removal to positions expressed in the site-to-site variable topocentric coordinate system (i.e. the intuitive North-East-Vertical system). After CME removal, we converted the coordinate system of the position to the local topocentric coordinate system (light blue time series in Fig. S7b).

Next, we removed the diurnal variation of the position time series expressed in the local topocentric coordinate system using a loess-based seasonal-trend decomposition method called STL^52,53^ (black time series in Fig. S7b). Here, the diurnal variation was estimated as the seasonal component, and we used the estimated trend and residual components for subsequent analysis to avoid dropping any short-term transient left in the residual component.

Then, we decimated the 30-s interval position time series into a 30-min interval (blue and pink time series in Fig. S7b) after applying the low-pass filter with a cut-off period of 1 h (yellow time series in Fig. S7b). This decimation was required to reduce computational burden for the subsequent slip inversion. As the time series to be filtered contain the permanent offset due mainly to the SSE, we applied a Hanning window with a width of 1 day at both ends of the time series (i.e. DOYs 073 and 103) prior to low-pass filtering using Seismic Analysis Code (SAC)^54,55^. After the low-pass filter was completed, we omitted the epochs used for tapering. We confirmed that no tremors were detected during these 2 days. In this decimation step, we also omitted epochs purely predicted by the Kalman filter—smoother (i.e. epochs at which no raw observables were available) during the kinematic positioning analysis.

Even after these data cleaning processes, we identified spurious outliers in the position time series, so we removed some of them satisfying the following criterion:

1 $$\left| {u_{i} - \frac{{q_{1} + q_{3} }}{2}} \right| > n*\frac{{q_{3} - q_{1} }}{2}$$

where *u*_*i*_ is the displacement at the *i*th epoch, *q*_1_ and *q*_3_ are the 25 and 75 percentile values of the position time series, respectively, and *n* is a threshold controlling how strict or loose we impose the outlier criterion (blue dots in Fig. S7b). We tested some different values for the threshold and finally preferred *n* = 5 to avoid removing the transient pattern likely representing the SSE-induced site motion, which means we removed epochs that deviated significantly from the main trend. We kept epochs where both the east and north components did not satisfy this criterion (pink time series in Fig. S7b).

### Spatiotemporal slip inversion

We employed a Kalman-filter-based inversion scheme which allows non-parametric inference of temporal slip evolution^40^. The forward model we used is described in Fukuda et al.^39^ and Fukuda^56^, except for a hyperparameter, *α*^2^, which controls the temporal smoothness of slip and slip rate; in this study we set *α*^2^ to be time-invariant. We inverted the two horizontal components of the cleaned position time series and did not use the vertical component because there is a significant trade-off between the vertical position and the zenith tropospheric delay estimates during the kinematic GPS analysis. We modelled the Juan de Fuca slab interface down to 60-km depth using Slab2 model^37^. The slab surface was tessellated by triangular subfaults (Fig. S16), and we computed the elastic deformation in response to their unit slip in the relative convergence direction of the converging plates (Fig. 1a)^57^ using a homogeneous elastic half-space model^58^. For simplicity, we ignored the elastic heterogeneity^59,60^. To alleviate the non-uniqueness of the spatial slip pattern and stabilise the inversion, we imposed a spatial smoothness constraint on the slip distribution. In this study, we took a singular value decomposition approach of Green’s functions to create a set of smooth basis functions^39,61^ and determined their amplitudes in the inversion. In this approach, the spatial smoothness of the estimated slip is controlled by the number of basis functions, *M*, and we can separately control the spatial and temporal smoothness. The temporal smoothness is controlled by one time-invariant hyperparameter *α*^2^ controlling the range of slip rate change from one epoch to the next^39,40,56^. With larger *M* and *α*^2^, the resultant spatial and temporal slip distributions become rougher, respectively (Fig. S6). We simultaneously modelled the site-specific random-walk motion of the benchmark^40^ and translation of the entire network, which were controlled by one scale factor for each^39,56^. Although we assumed that a substantial portion of the CME had already been removed, we included the latter term in the slip inversion as well because our CME removal by the post-processing was based on the “estimation” of ICA, which potentially missed some CME. Typically, these four hyperparameters, namely, *M*, *α*^2^, and scale factors for the random-walk and the translation components, need optimising by maximising the likelihood^39,40,56^. However, in this study, we assumed $1\,{\text{ mm}}/\sqrt {{\text{year}}}$ and 5 mm for the scale factors of random-walk^56^ and the translation components, respectively. We first attempted to determine *M* and *α*^2^ by maximising the likelihood, but this resulted in an extremely rough unphysical spatiotemporal slip distribution (Figs. S5 and S6). Hence, we determined the preferred combination of *M* and *α*^2^ to be 18 and 1000 m^2^/year^3^, respectively, by inspecting an inflexion point of *α*^2^ versus log-likelihood curves with various *M* (Fig. S5). Here, we assumed that a gentler increase in log-likelihood beyond the preferred *M* and *α*^2^ reflected data fitting improvement by describing the unmodelled enigmatic signature in the data. We present four test models with different *M* and *α*^2^ values, which demonstrate rougher or smoother spatiotemporal slip patterns in Fig. S6. The failure of maximum-likelihood determination of these hyperparameters indicates that the formal covariance matrix does not reasonably estimate the uncertainty for slip and slip rates. Hence, we preferred not to provide formal estimation errors for our results and exclusively discussed the obvious features.

We applied the same inversion scheme to the daily GPS coordinates provided by NGL^50^. We converted the daily coordinates to those relative to DRAO to realise the same reference frame as the kinematic coordinates (Figs. 4b,d,f and 5 and Videos S3 and S4). We used the hyperparameters determined by maximising the log-likelihood because it yielded the physically plausible spatiotemporal slip evolution. The same number of the basis functions, *M* = 18, as the preferred model of the subdaily inversion yielded the maximum log-likelihood in the daily inversion. The temporal smoothness parameter is estimated to be *α*^2^ = 1778.28 m^2^/year^3^. The inferred model reasonably fits the daily GPS coordinates (Figs. S17–S19).

### Moment and moment rate

We computed the moment and moment rate of the inferred SSE by integrating the estimated slip and slip rate, respectively, over all the subfaults (Fig. S16), including those with a negative slip or slip rate. The rigidity value was set to 30 GPa.

## Supplementary Information

Supplementary Information.

Supplementary Video S1.

Supplementary Video S2.

Supplementary Video S3.

Supplementary Video S4.

## Data Availability

The RINEX files were retrieved via UNAVCO (https://data.unavco.org/archive/gnss/rinex/obs/) and the Western Canada Deformation Array (WCDA; ftp://wcda.pgc.nrcan.gc.ca) servers. FES2004 ocean tide model is available at http://holt.oso.chalmers.se/loading/. TRACK in GAMIT/GLOBK program^44–46^ and Seismic Analysis Code (SAC)^54,55^ are available upon request to the developers at http://geoweb.mit.edu/gg/license.php and http://ds.iris.edu/ds/nodes/dmc/forms/sac/, respectively. We made our slip and slip rate files available at Zenodo as Itoh et al. ^63^.
