# Supplementary material for: Imaging evolution of Cascadia slow-slip event using high-rate GPS
Source: Sci Rep. 2022 May 3;12:7179. doi: 10.1038/s41598-022-10957-8 (PMC9065071; doi:10.1038/s41598-022-10957-8)
Supplement: Supplementary file 1 — Supplementary Information. [file 41598_2022_10957_MOESM1_ESM.pdf]

Supplementary Information

Imaging evolution of Cascadia slow-slip event by high-rate GPS

Yuji Itoh[1, 2, *], Yosuke Aoki[1], Junichi Fukuda[1]

[1]Earthquake Research Institute, the University of Tokyo, Japan
[2]Now at Institut des Sciences de la Terre, Université Grenoble Alpes, France.

[*]Corresponding author: Yuji Itoh (yuji.itoh@univ-grenoble-alpes.fr)

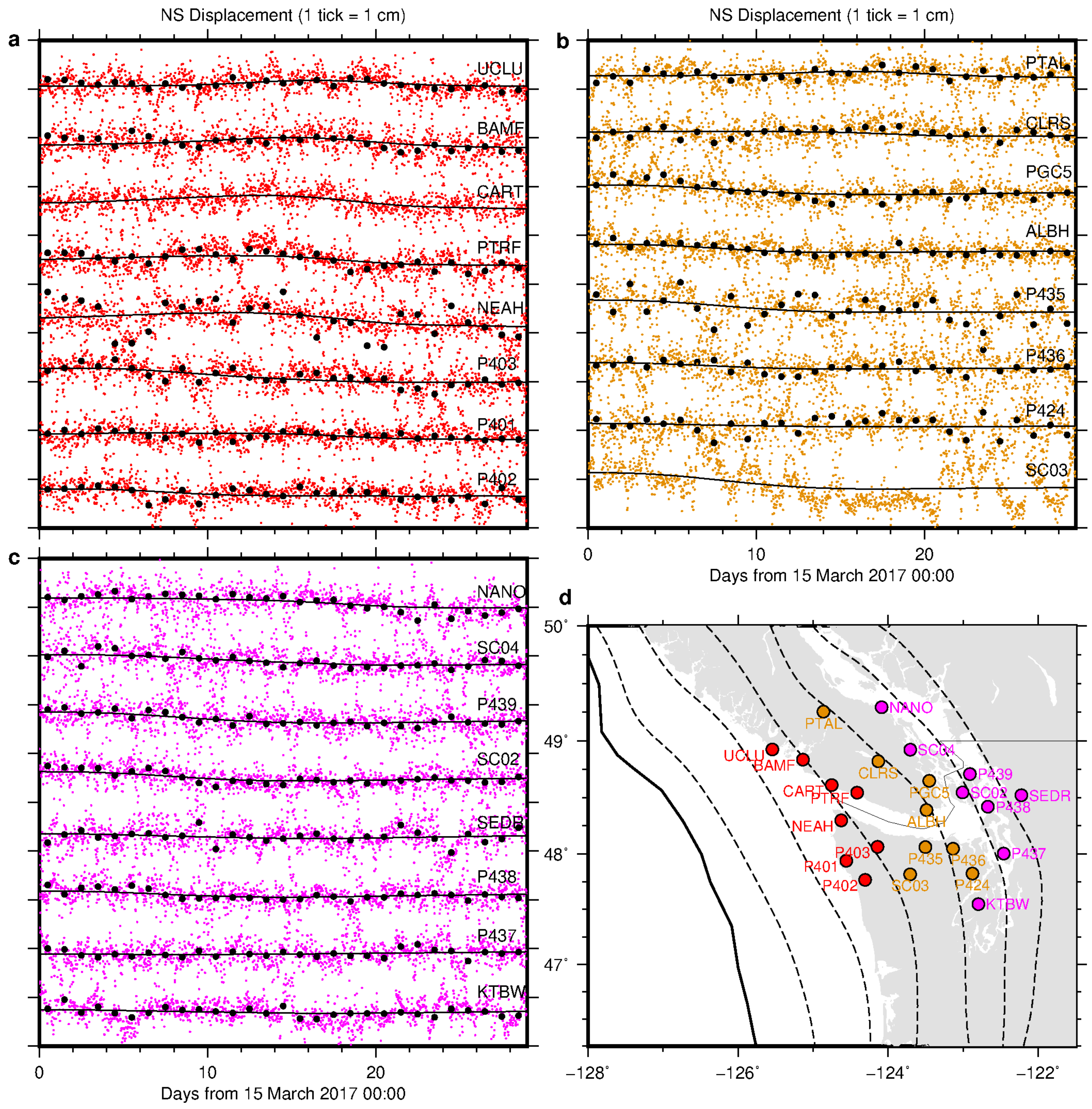

Figure. S1. Kinematic GPS data at a 30-minute interval and fitting by spatiotemporal slip model. **a** – **c**. The coloured dots indicate the post-processed north component of kinematic 30-min GPS positions, further corrected for whole network translation estimated in 30-min slip inversion (see Method). The overlying black dots indicate daily static east component which were corrected for whole network translation estimated in the daily slip inversion (see Method). The daily solutions happen to be unavailable at CART and SC03. The overlying black solid lines indicate the predicted motion due to the fault slip. **d**. Site location. The coloured circles indicate the site location of the time series with the same colour in **a** – **c**.

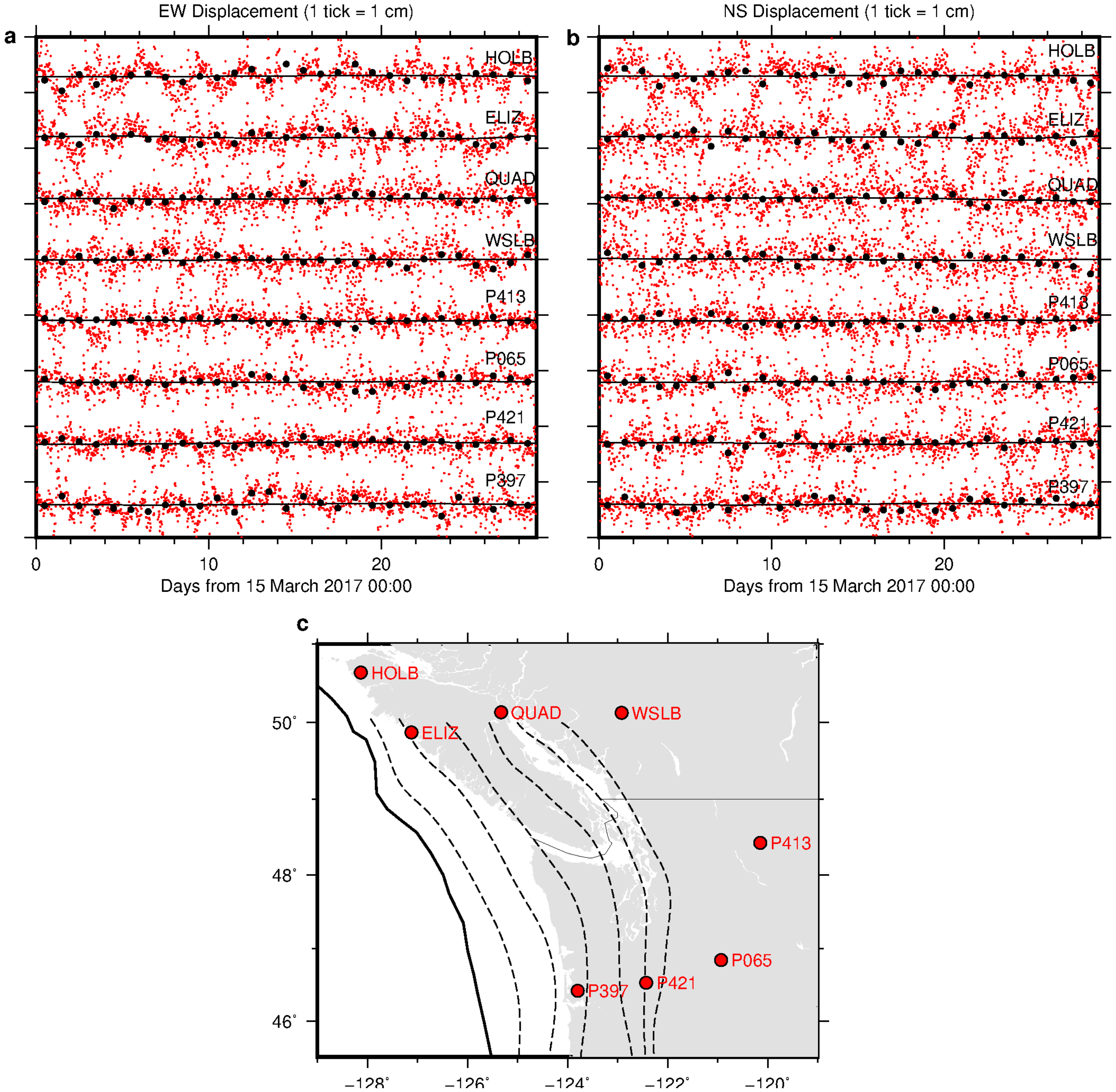

Figure. S2. Kinematic GPS data (30-minute interval) at distant sites from the area of the SSE of interest and fitting by spatiotemporal slip model. **a** – **b**. The red dots indicate the post-processed east (**a**) and north (**b**) components of kinematic GPS positions, which are further corrected for whole network translation estimated in slip inversion (see Method). The overlying black dots indicate daily static east component which were corrected for whole network translation estimated in the daily slip inversion (see Method). The overlying black solid lines indicate the predicted motion due to the fault slip. **c**. Site location. The red circles indicate the site location of the time series in **a** – **b**.

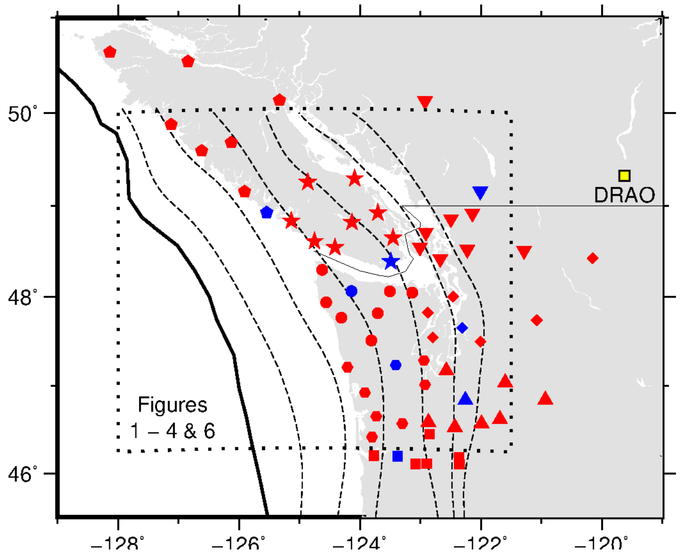

Figure. S3. Group of kinematic sites. Sites in each of the eight groups are indicated by different symbols (red and blue). The blue symbols indicate the reference sites used for kinematic GPS analysis of the sites in each group (red). The yellow square indicates DRAO, the reference site of the entire network. The box with dots indicates the area shown in Figures 1 – 4 and 6.

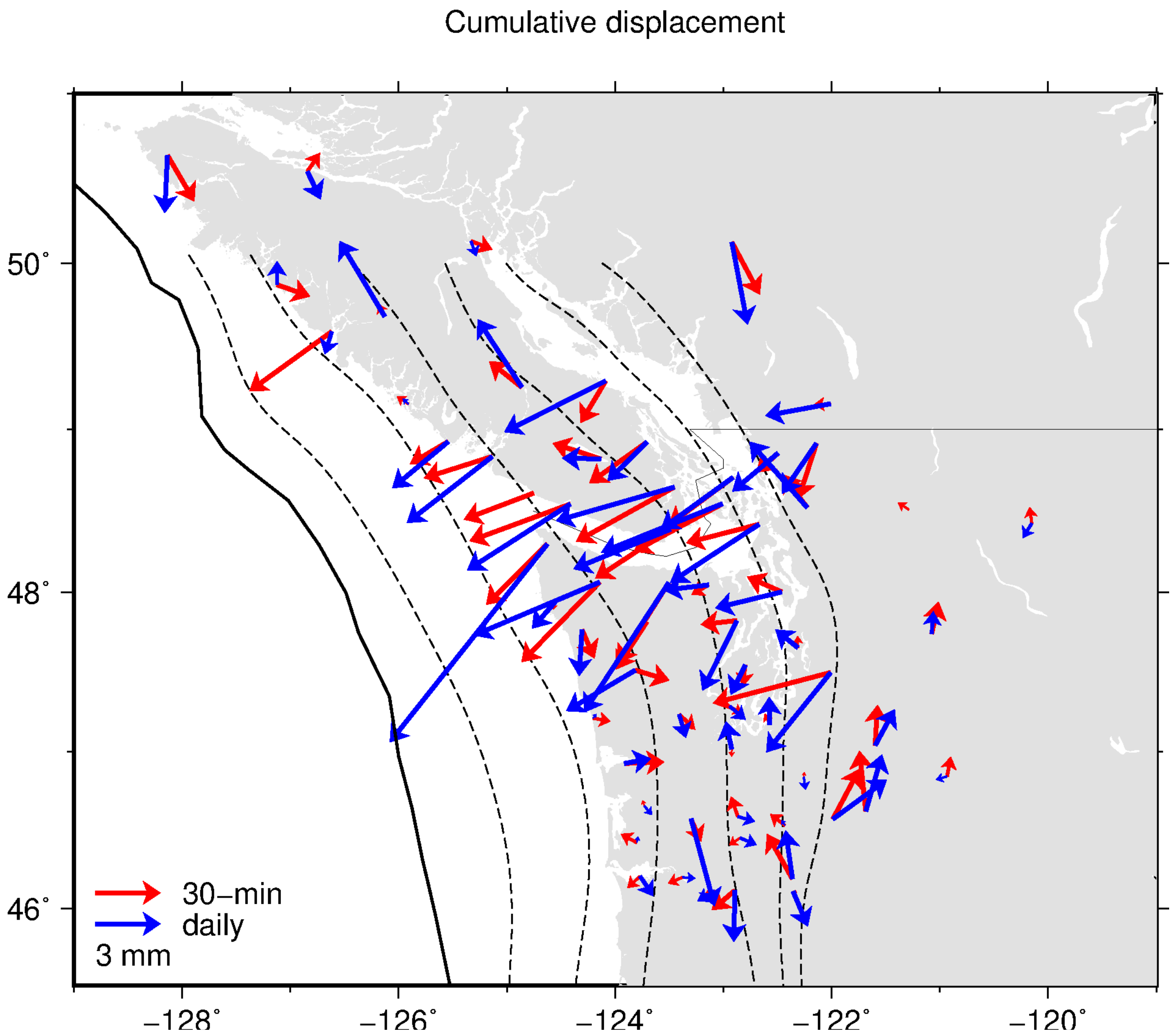

Figure. S4. Cumulative GPS displacements using the 30-min (red) and the daily (blue) data at all the sites.

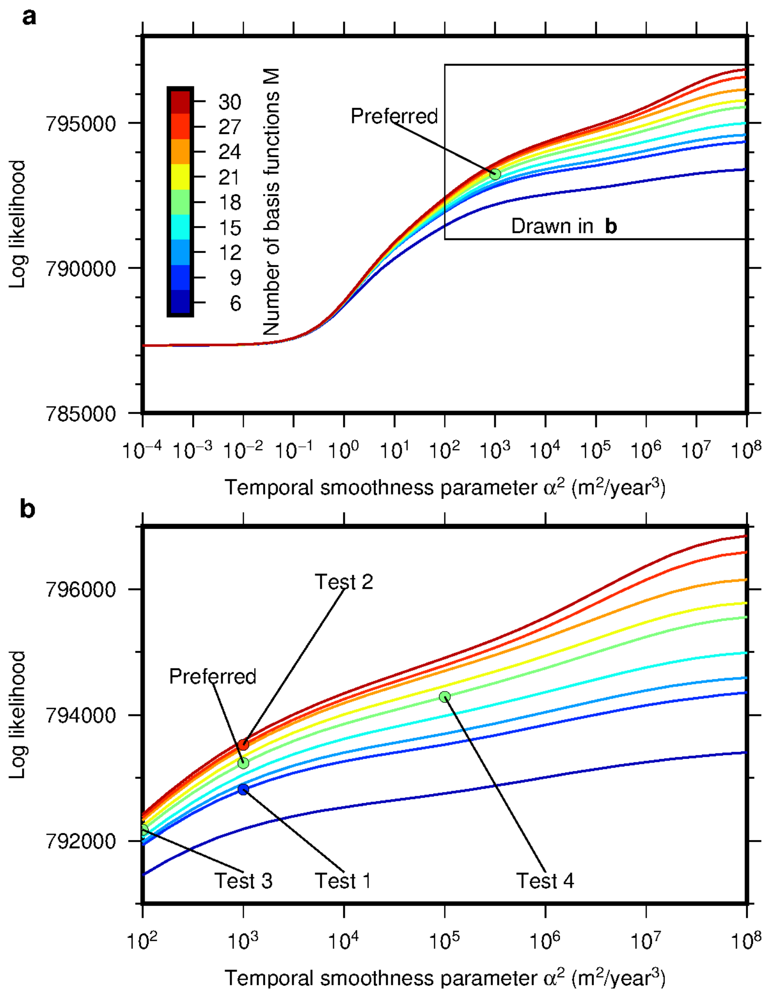

Figure. S5. Diagram of log-likelihood with various hyperparameters. **a**. The coloured solid lines indicate log-likelihood values with different temporal roughness parameters α^2. Each colour indicates the number of basis functions, M. The box with the solid line indicates the region of **b**. The green dots indicate the preferred model as labelled. **b**. Zoom-in plot of log-likelihood curves with a large α^2. The coloured dots indicate the preferred (Figures 4a, 4c, 4e, 5 and 6) and test models (Figure S6) as labelled.

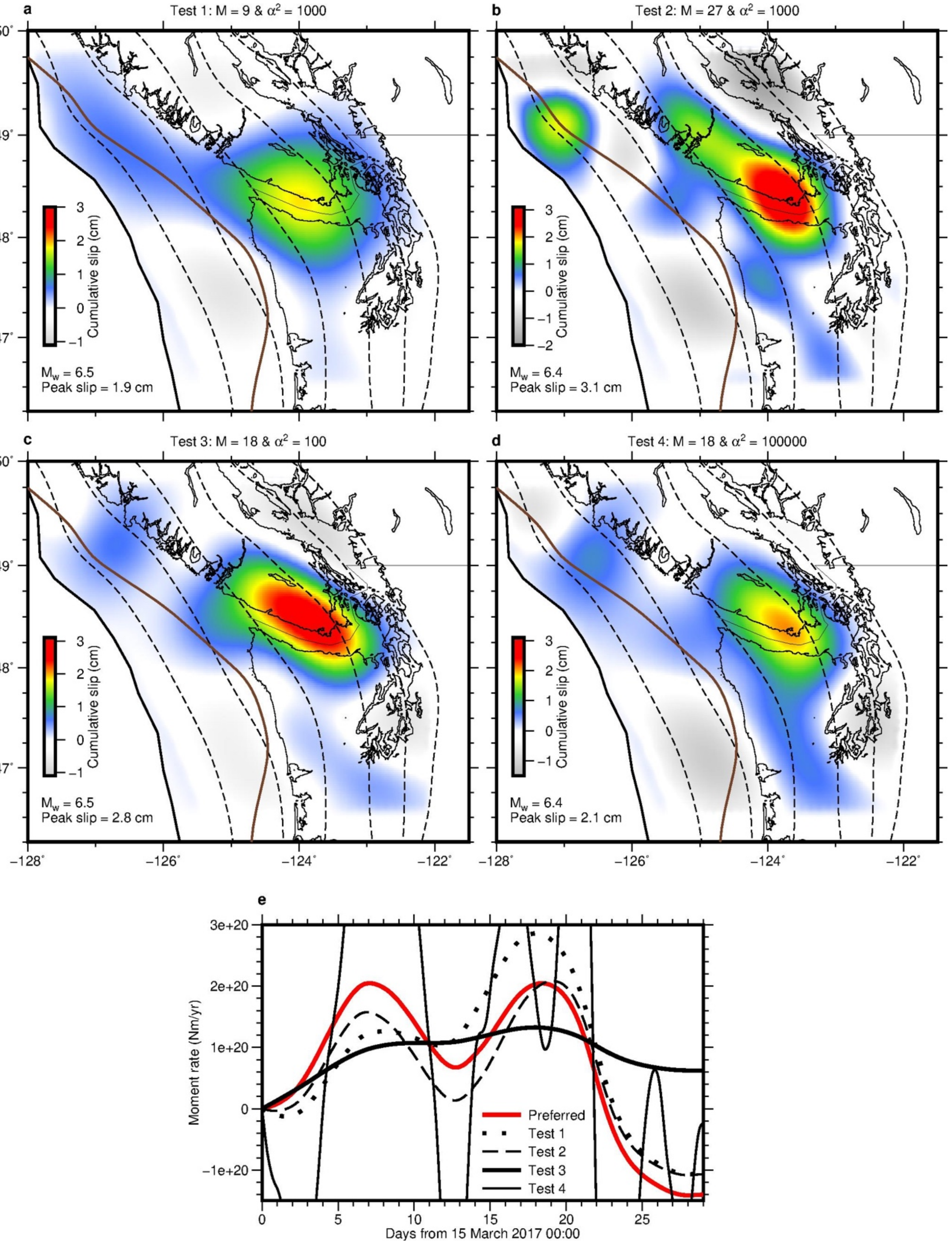

Figure. S6. Test models with different hyperparameters for spatiotemporal smoothness of slip. **a** – **d.** Cumulative slip with different numbers of basis functions M and temporal smoothness parameter α^2 in $m^2/year^3$ as labelled. **e**. Moment rate history of preferred and test models. Curves with different line types indicate different models, as shown in the legend. The curve for test 4 (thinner solid black line) was saturated.

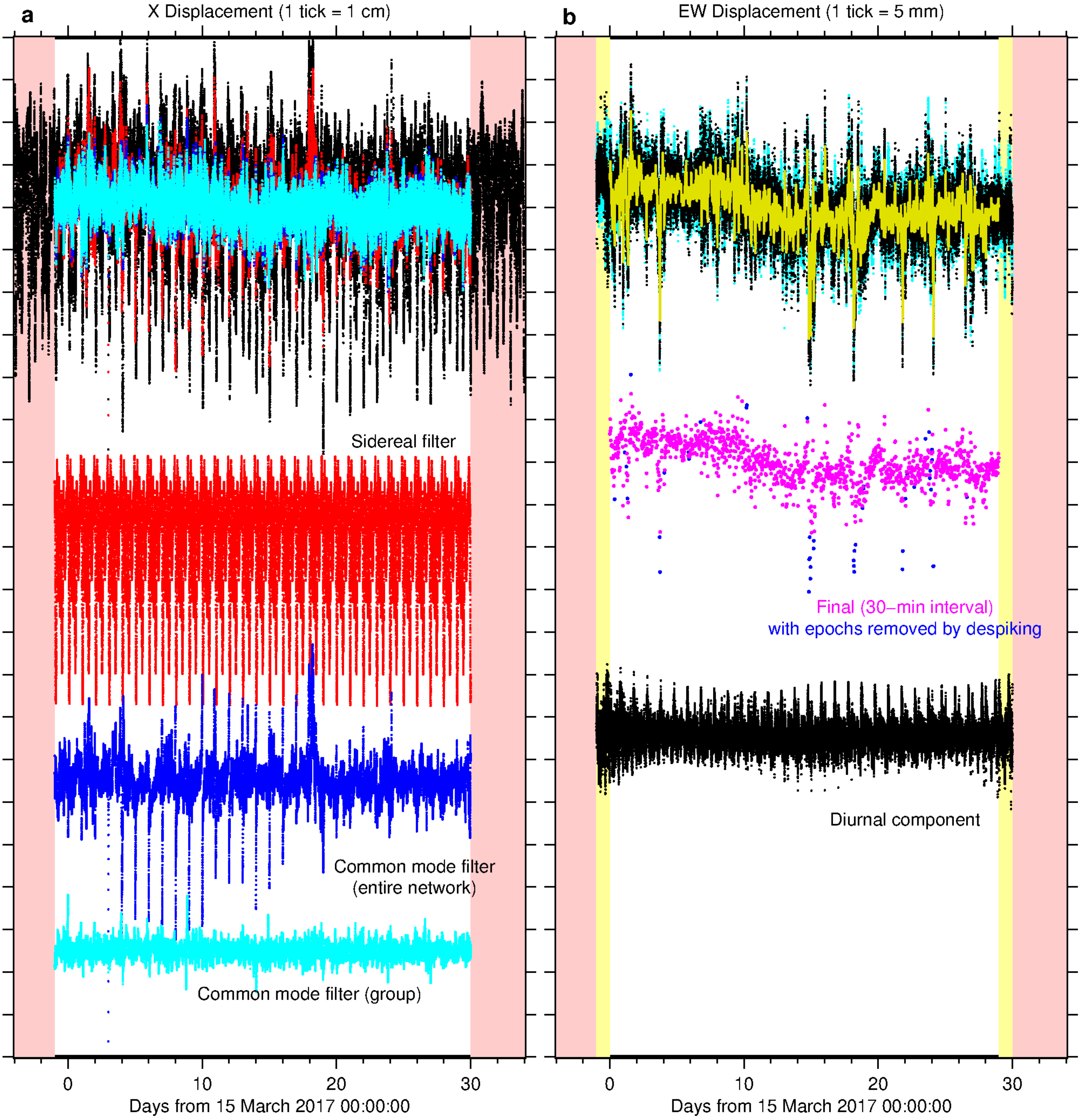

Figure S7. Example of post-processing procedure of kinematic GPS position time series at a 30-second interval. **a**. Displacement in the X-direction in the geocentric coordinate system at ALBH. The top time series indicate the original (black), sidereal-filtered (red), first-common-mode-filtered (blue), and second-common-mode-filtered (light blue) position time series. The filters used for these steps were plotted with the corresponding colours as labelled. The light red background highlights the data period at both ends which are used to construct the sidereal filter. **b**. Displacement in the EW direction at the ALBH. The top time series indicate the common-mode-filtered (light blue), diurnal-component-removed (black), and low-pass filtered (yellow) position time series. The middle blue and purple time series indicate the decimated position time series with an interval of 30 min, from which the epochs indicated in blue were omitted by despiking to obtain the final position time series (purple). The bottom black time series indicates the estimated diurnal components. The yellow background highlights the data period at both ends which are used to taper.

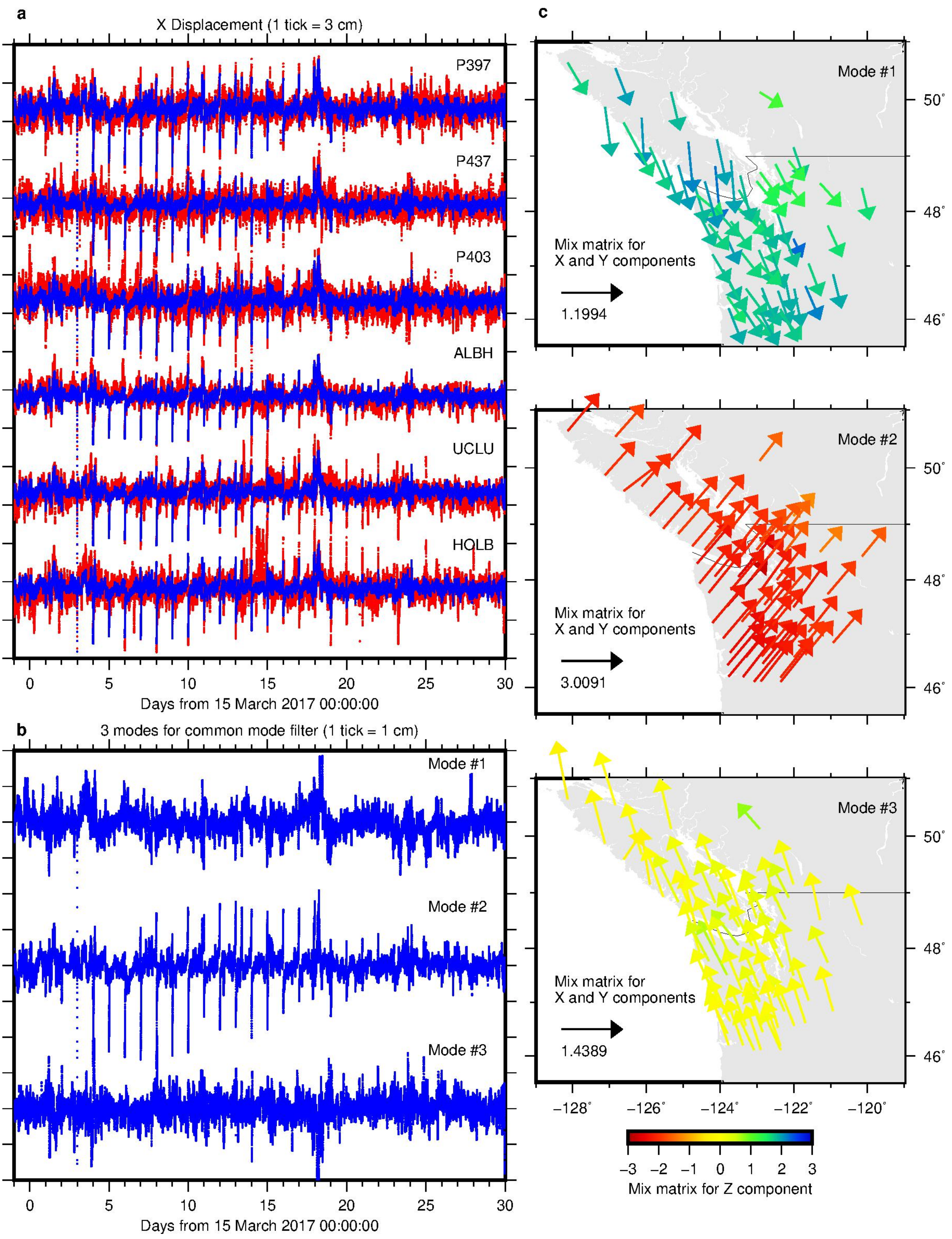

Figure S8. ICA-based common-mode filtering for the entire network **a**. Examples of post-sidereal-filtered position time series in the X-direction in the geocentric coordinate system at selected sites as labelled (red) with common-mode filter estimated at each site (blue). Site locations are shown in Figures S1 and S2. **b**. Three independent components (modes) representing the common-mode error estimated by the ICA. **c**. Spatial pattern (i.e., mix matrix) for the three modes shown in B. East and north components of the vectors indicate X and Y components in the geocentric coordinate system of the mix matrix at each site, respectively, with their Z components color-coded.

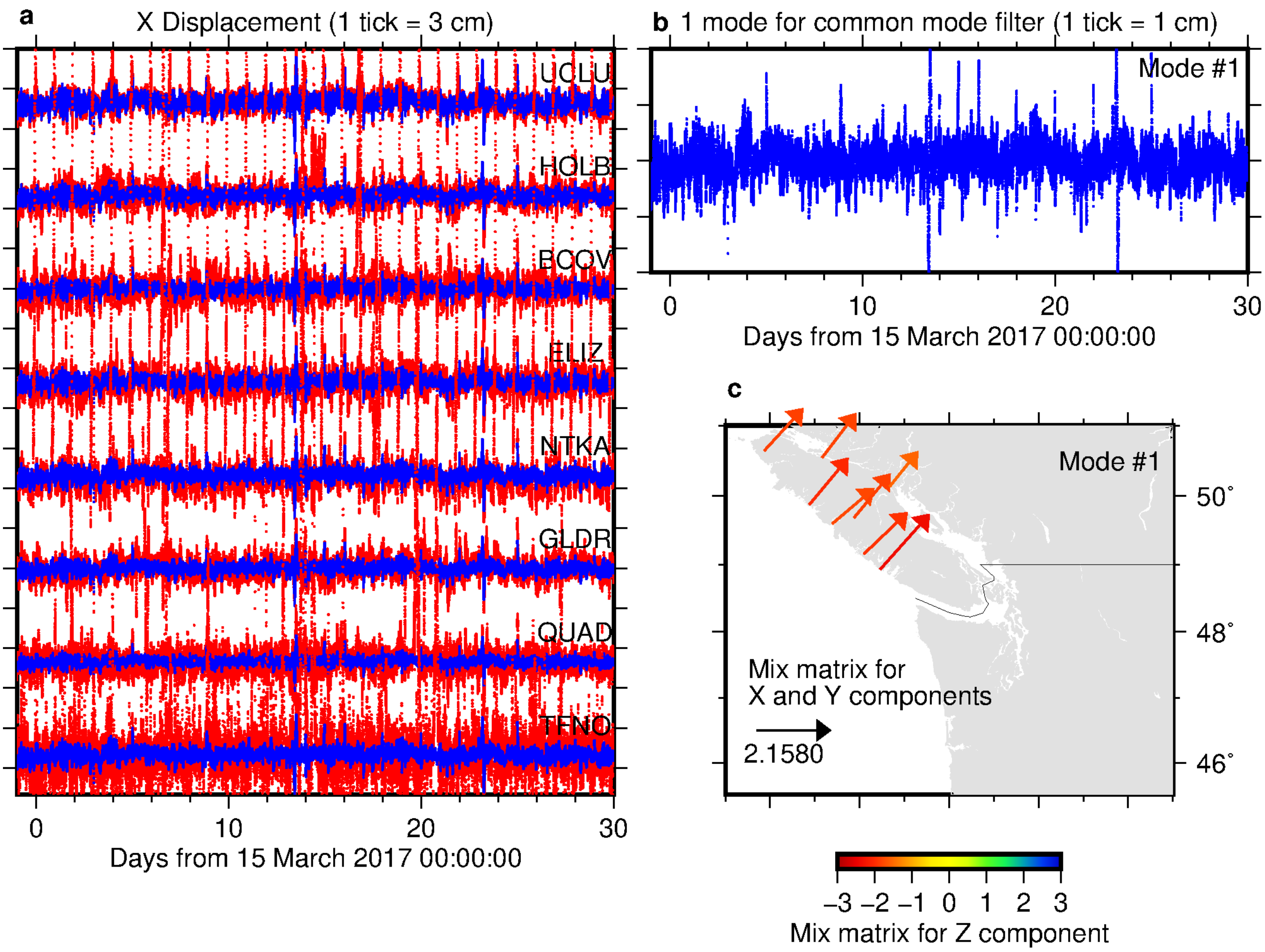

Figure S9. ICA-based common-mode filtering for one of the eight subnets. The red time series in **a** indicate the time series after the first common-mode filtering (i.e., Figure S8). See Figure S8 for the other legends.

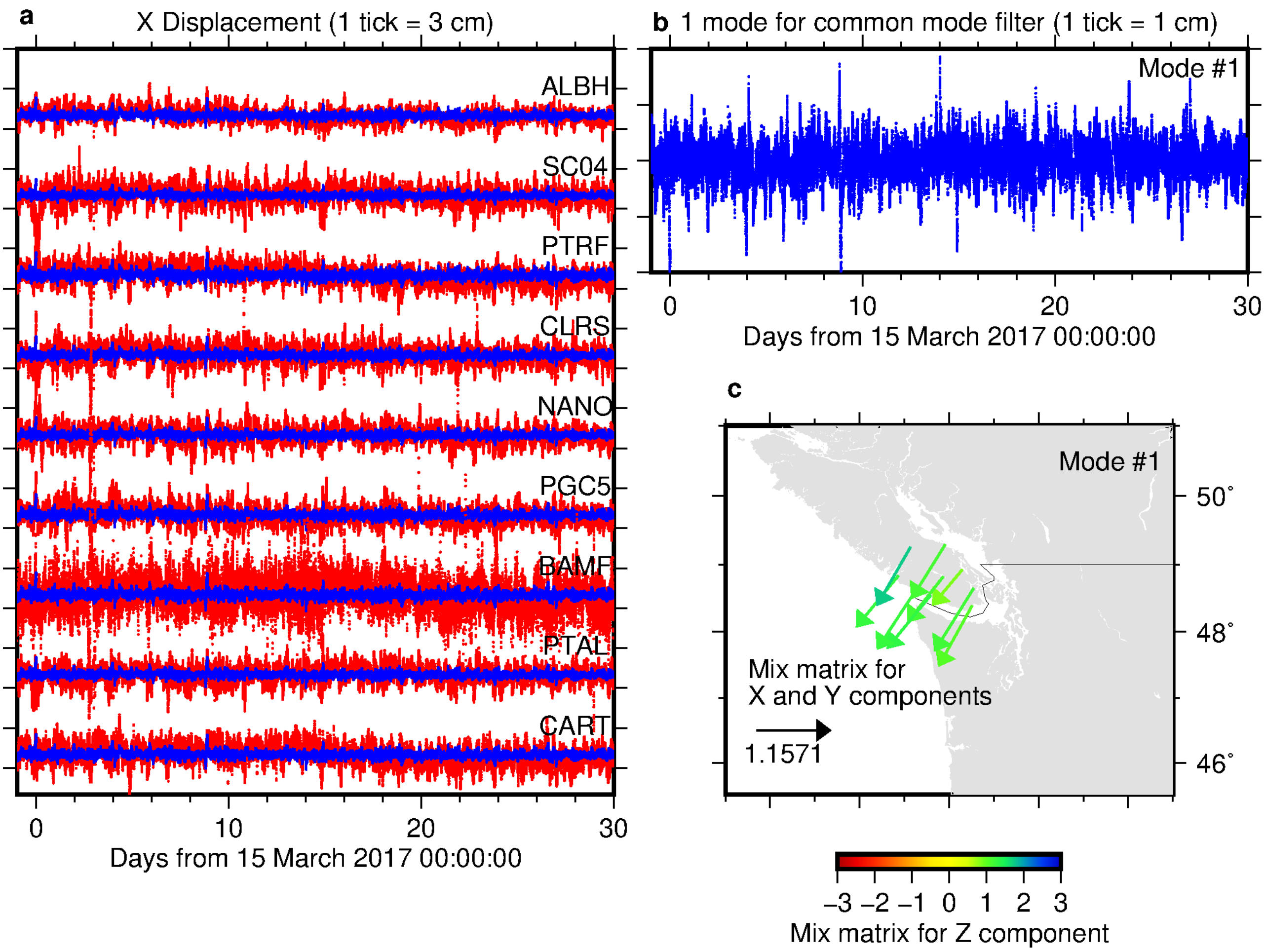

Figure S10. ICA-based common-mode filtering for one of the eight subnets. The red time series in **a** indicate the time series after the first common-mode filtering (i.e., Figure S8). See Figure S8 for the legends.

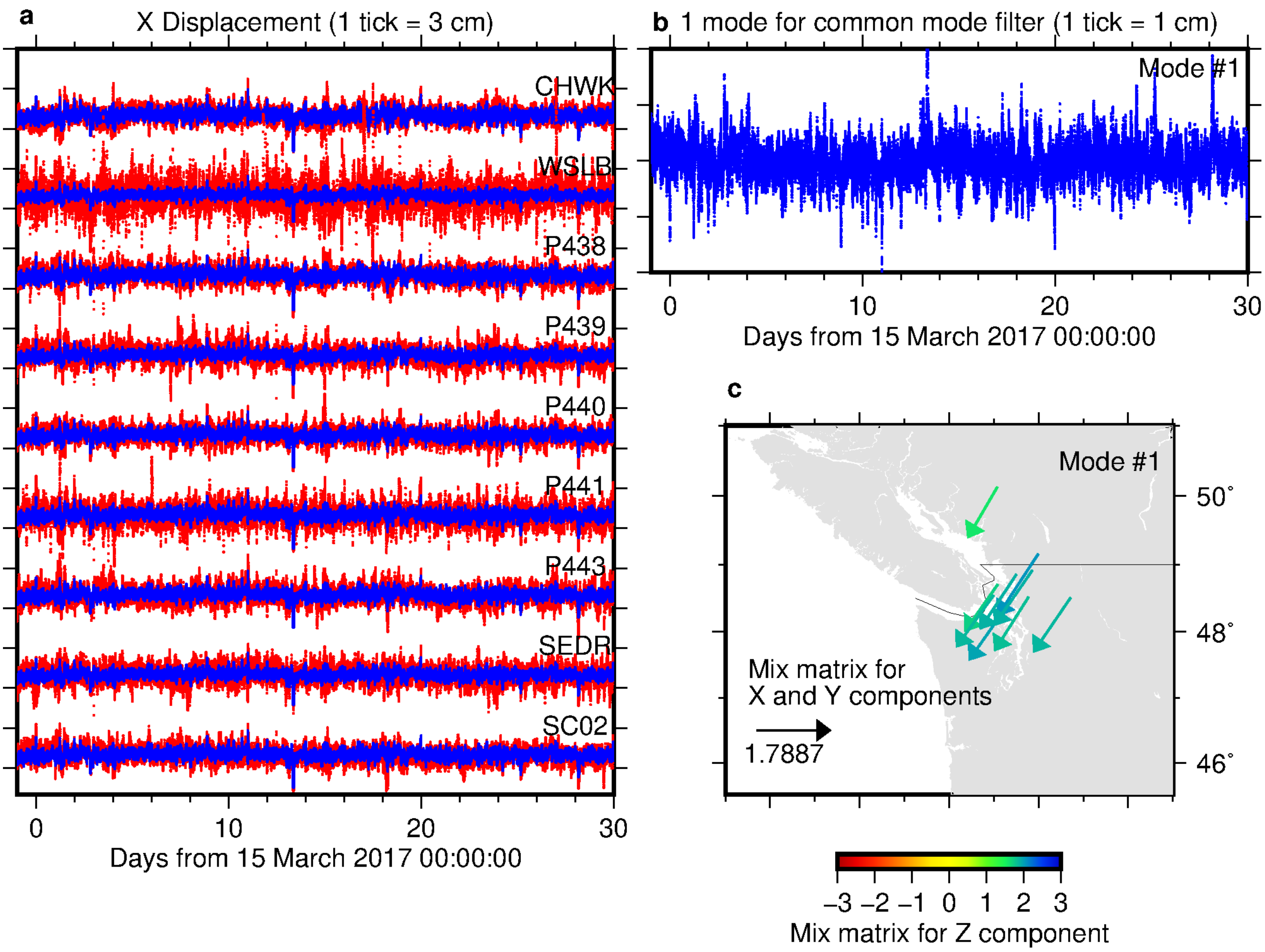

Figure S11. ICA-based common-mode filtering for one of the eight subnets. The red time series in **a** indicate the time series after the first common-mode filtering (i.e., Figure S8). See Figure S8 for the legends.

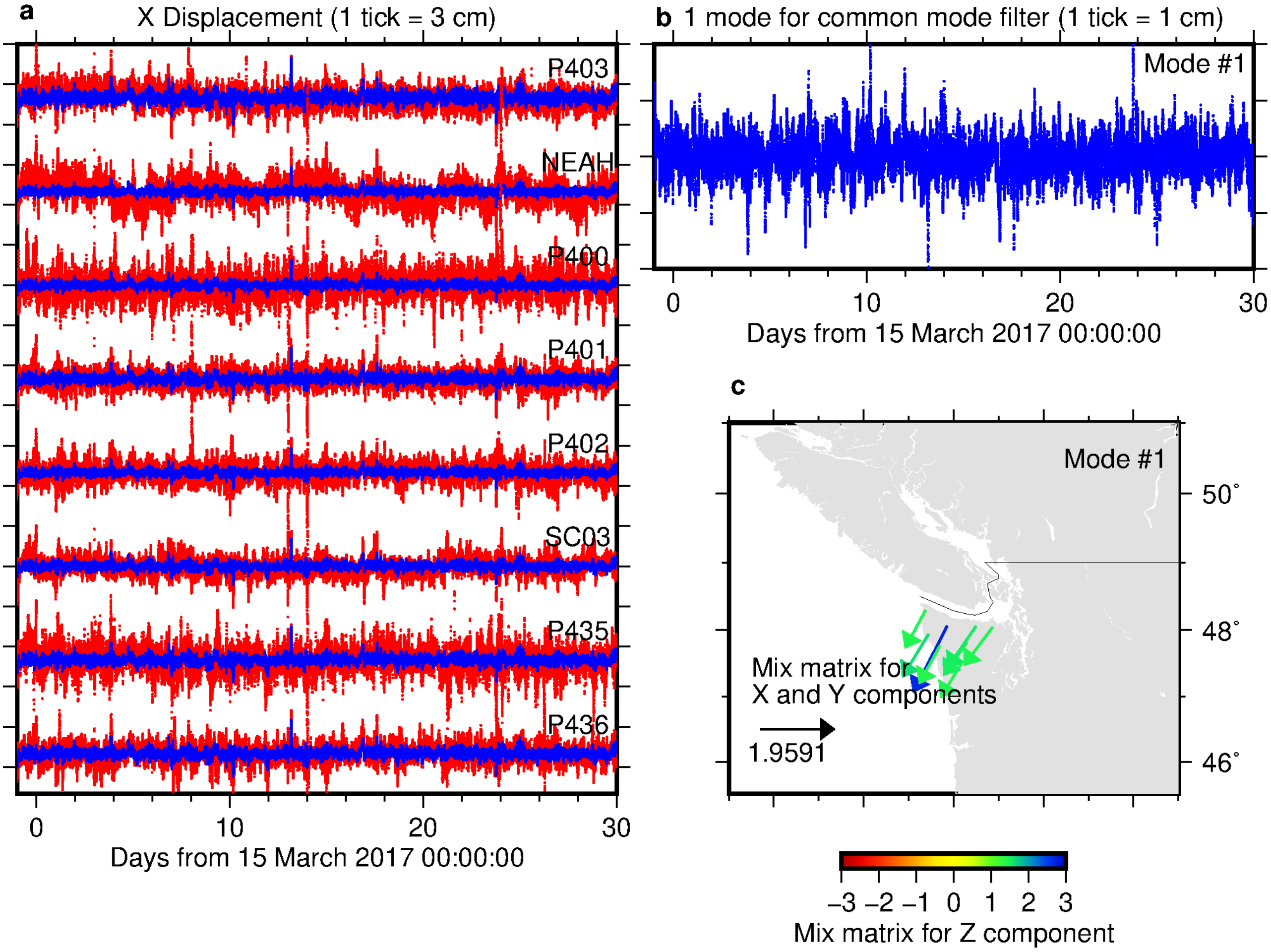

Figure S12. ICA-based common-mode filtering for one of the eight subnets. The red time series in **a** indicate the time series after the first common-mode filtering (i.e., Figure S8). See Figure S8 for the legends.

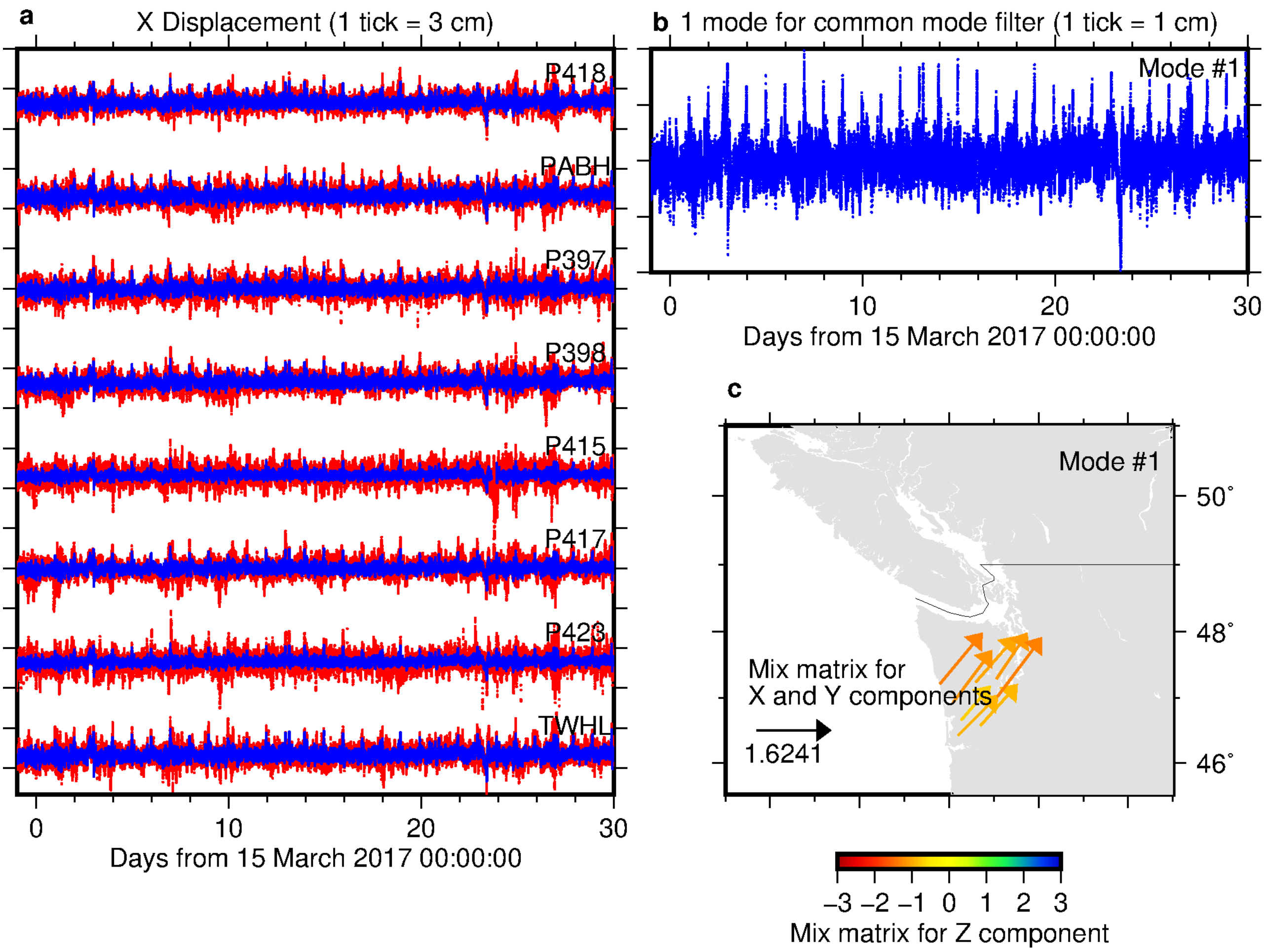

Figure S13. ICA-based common-mode filtering for one of the eight subnets. The red time series in **a** indicate the time series after the first common-mode filtering (i.e., Figure S8). See Figure S8 for the legends.

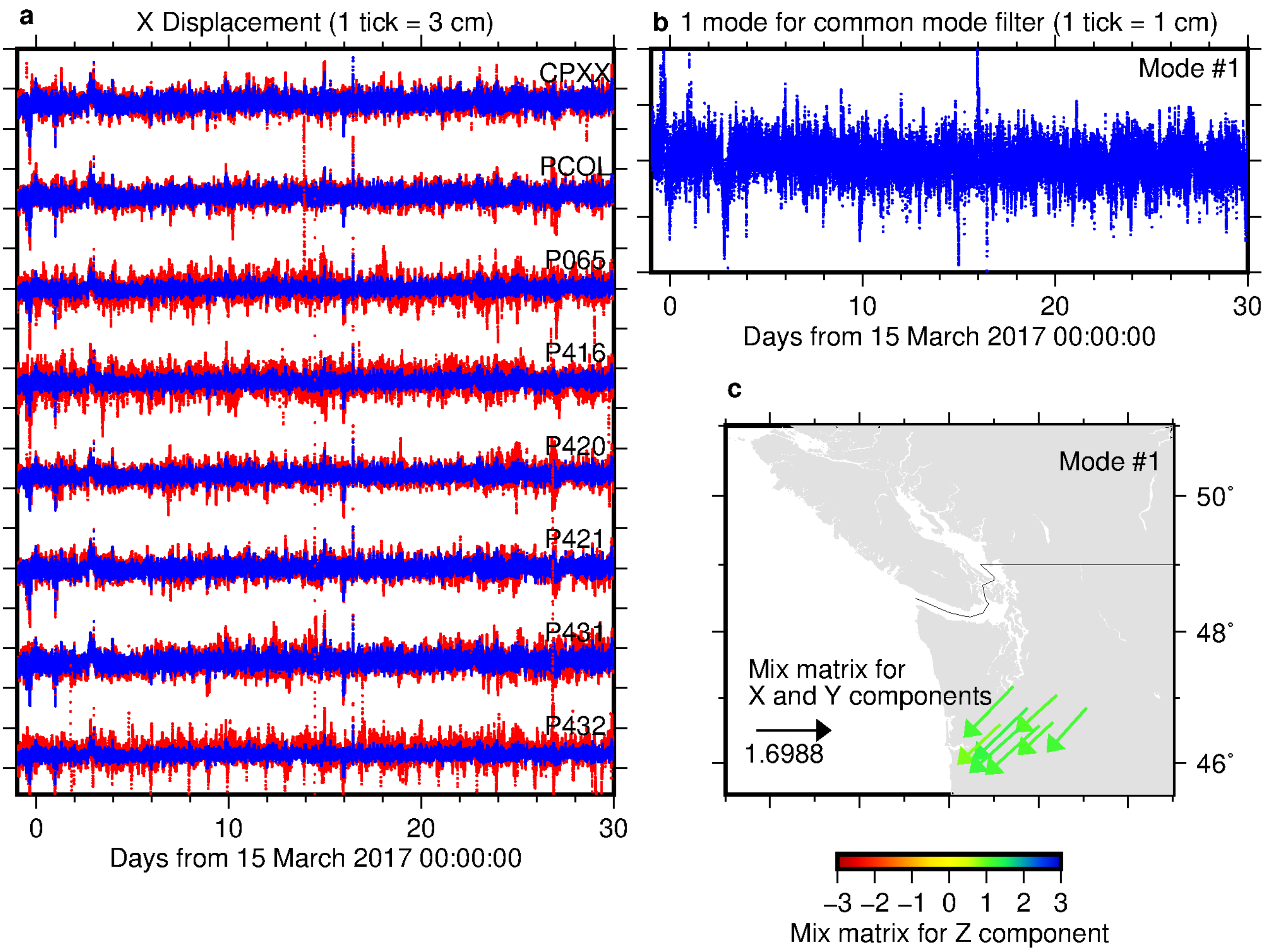

Figure S14. ICA-based common-mode filtering for one of the eight subnets. The red time series in **a** indicate the time series after the first common-mode filtering (i.e., Figure S8). See Figure S8 for the legends.

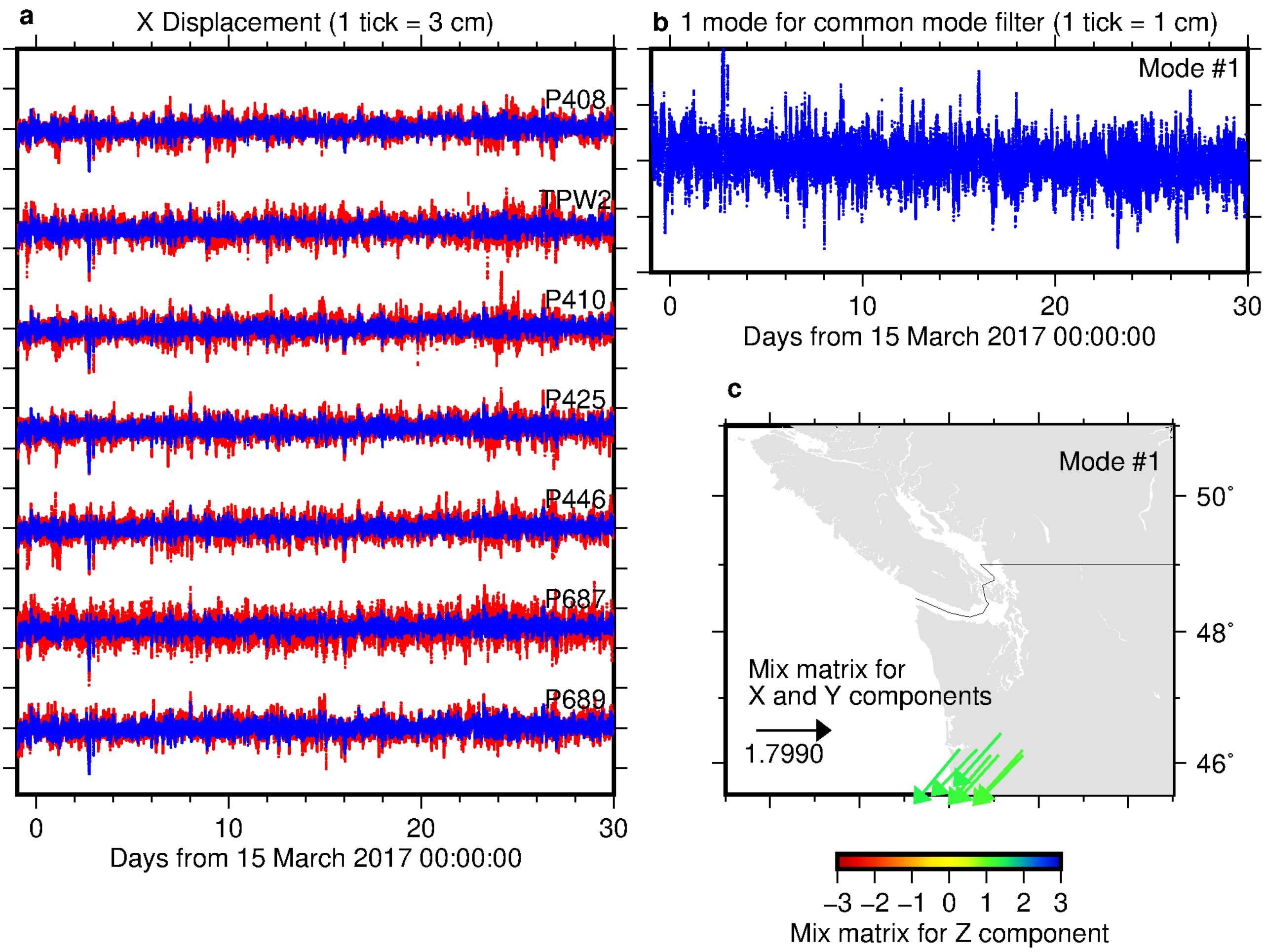

Figure S15. ICA-based common-mode filtering for one of the eight subnets. The red time series in **a** indicate the time series after the first common-mode filtering (i.e., Figure S8). See Figure S8 for the legends.

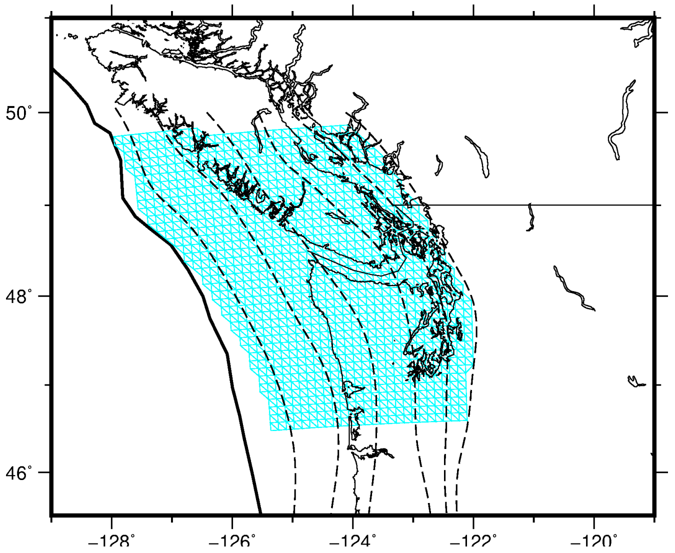

Figure S16. Fault configuration. The light blue triangles indicate the subfaults used to generate the elastic Green's functions.

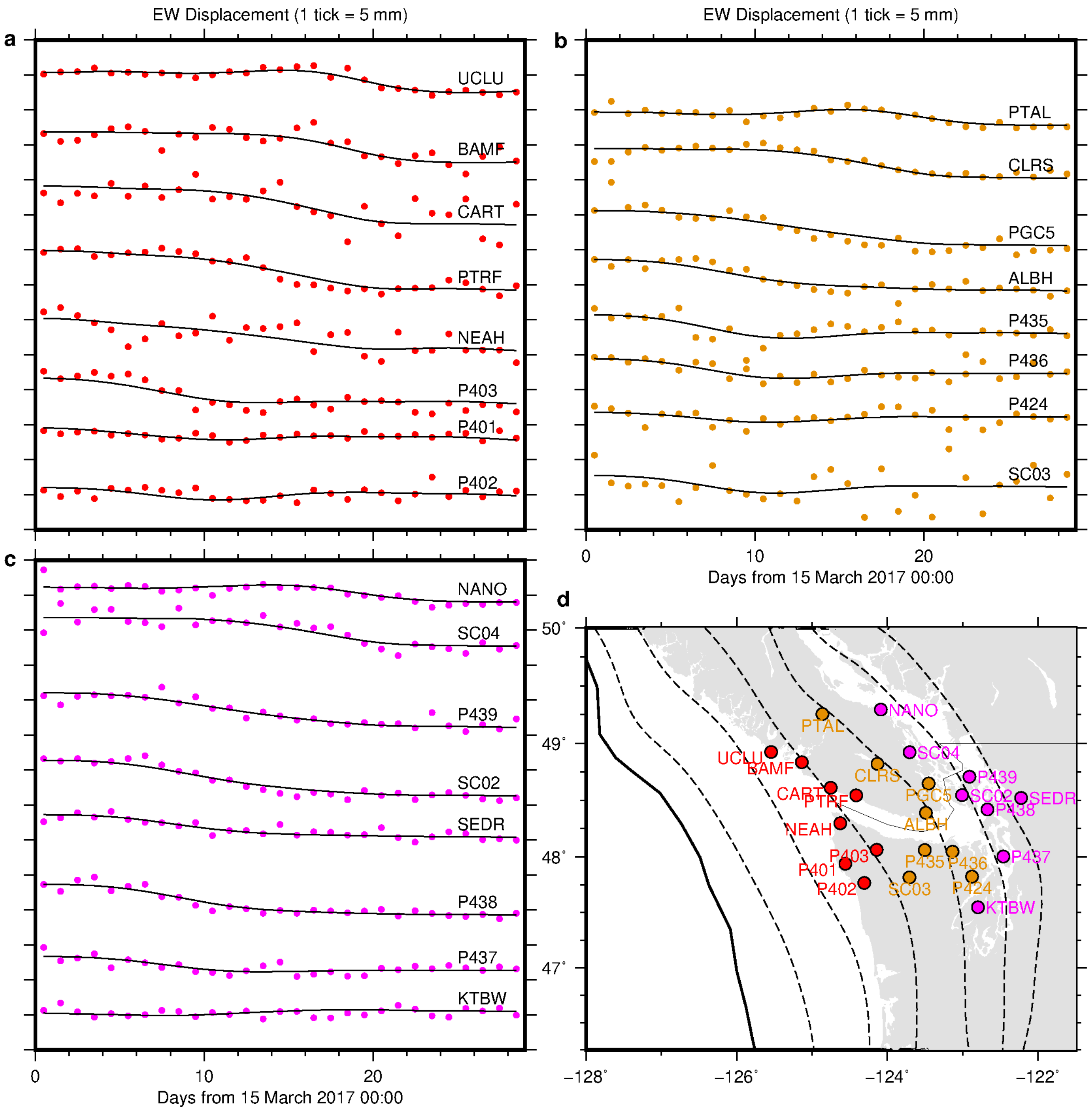

Figure. S17. Daily GPS data and fitting by spatiotemporal slip model. **a** – **c**. The coloured dots indicate east component of daily GPS positions, further corrected for whole network translation estimated in the daily slip inversion (Figure 4b, 4d and 4f; see Method). The daily solutions happen to be unavailable at CART and SC03, so we used the kinematic coordinates at every noon instead. The overlying black solid lines indicate the predicted motion due to the fault slip. **d**. Site location. The coloured circles indicate the site location of the time series with the same colour in **a** – **c**.

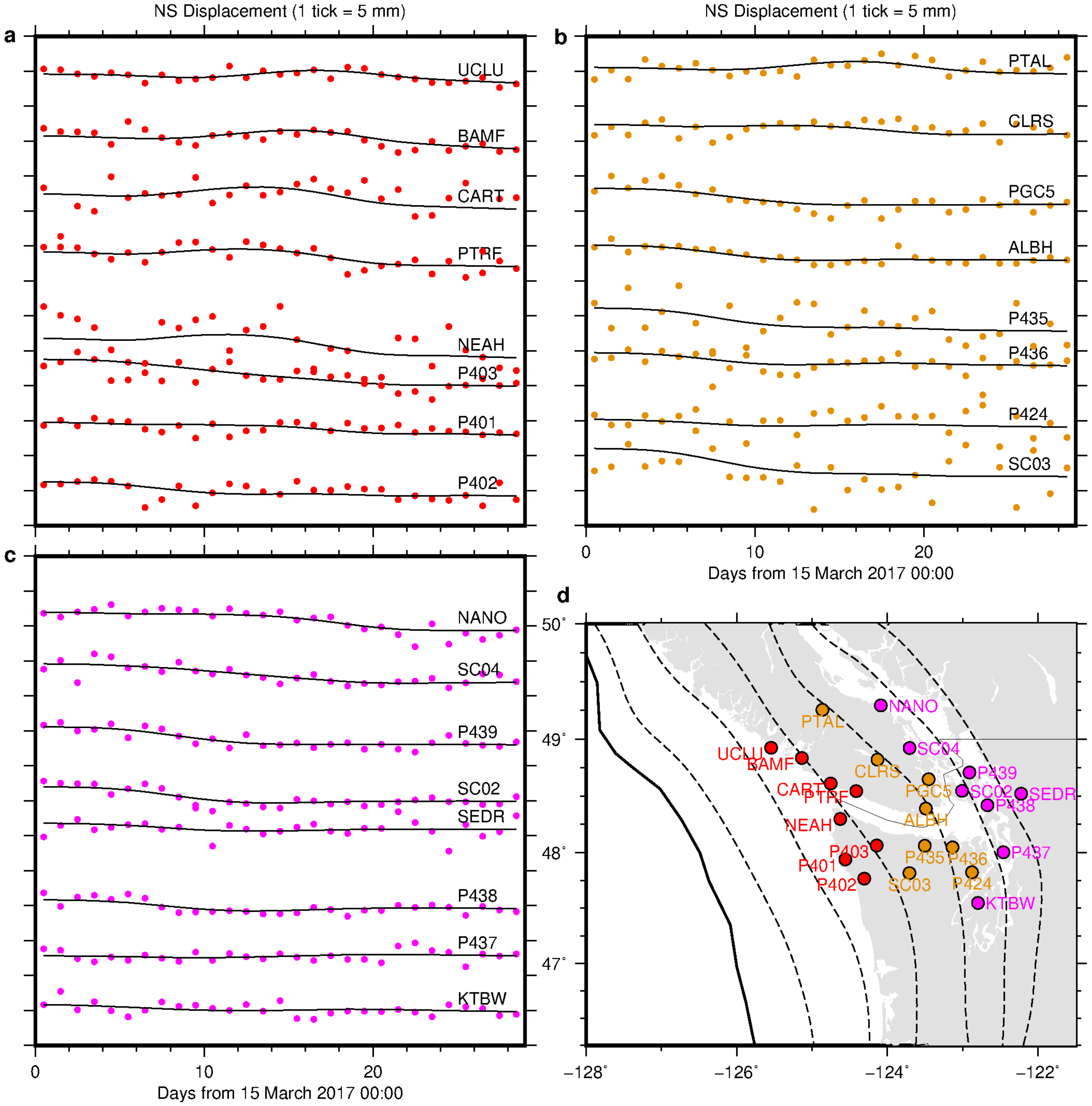

Figure. S18. Daily GPS data and fitting by spatiotemporal slip model. **a** – **c**. The coloured dots indicate north component of daily GPS positions, further corrected for whole network translation estimated in the daily slip inversion (Figure 4b, 4d and 4f; see Method). The daily solutions happen to be unavailable at CART and SC03, so we used the kinematic coordinates at every noon instead. The overlying black solid lines indicate the predicted motion due to the fault slip. **d**. Site location. The coloured circles indicate the site location of the time series with the same colour in **a** – **c**.

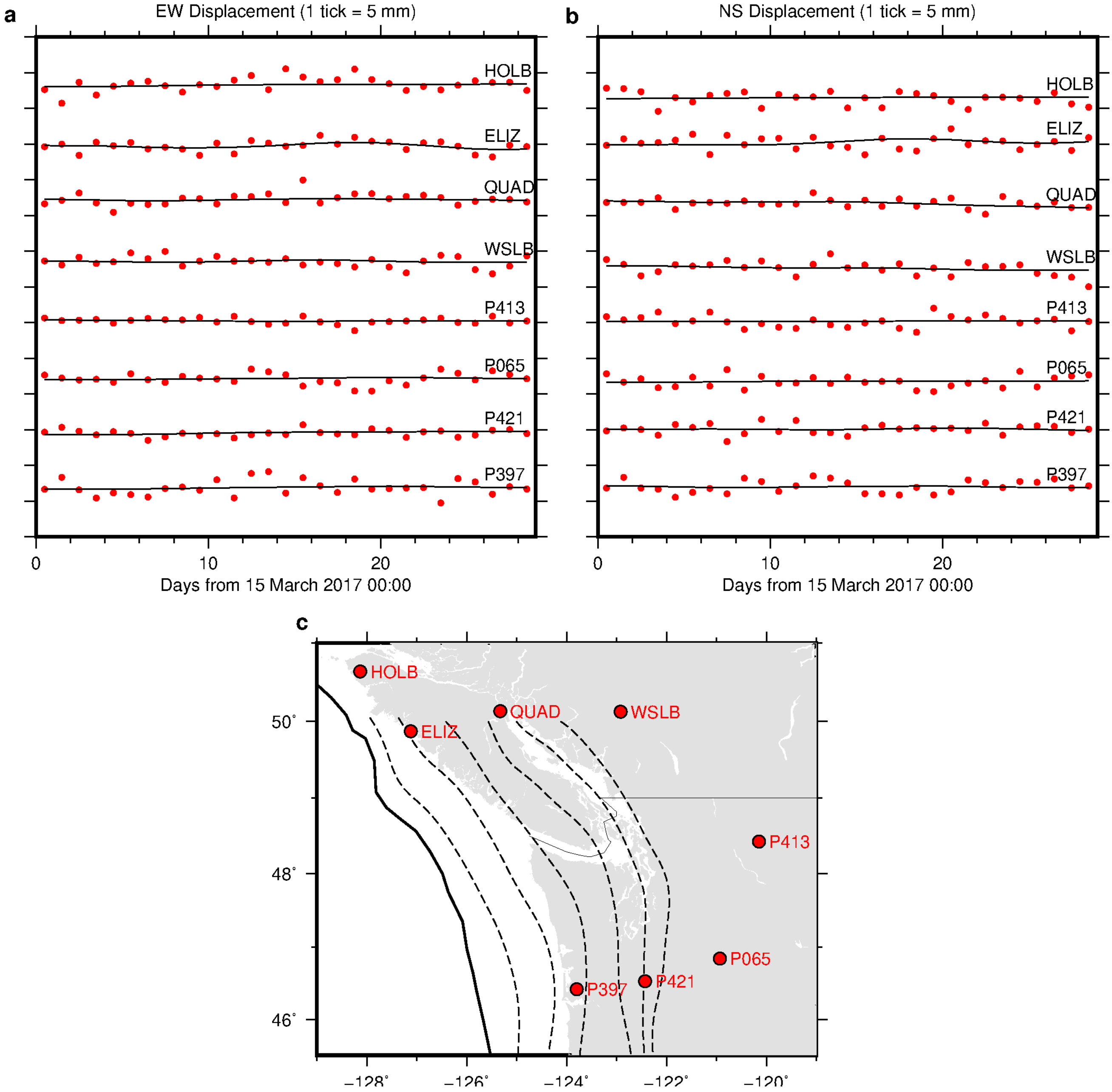

Figure. S19. Daily GPS data at distant sites from the area of the SSE of interest and fitting by spatiotemporal slip model. **a** – **b**. The red dots indicate east (**a**) and north (**b**) components of daily GPS positions, which are further corrected for whole network translation estimated in slip inversion (Figure 4b, 4d and 4f; see Method). The overlying black solid lines indicate the predicted motion due to the fault slip. **c**. Site location. The red circles indicate the site location of the time series in **a** – **b**.

Video S1.
Slip evolution over the entire period at a 30-minute interval. See Figure 4a for the legends.

Video S2.
Slip rate evolution over the entire period at a 30-minute interval. The purple dots indicate tremor epicentres during each 30-minute bin. See Figure 4a for the legends.

Video S3.
Daily slip evolution over the entire period obtained from the inversion of daily GPS data. See Figure 4b for the legends.

Video S4.
Daily slip rate evolution over the entire period obtained from the inversion of daily GPS data. The purple dots indicate tremor epicentres during a daily bin. See Figure 4b for the legends.
